# Semaphorin 4D in human head and neck cancer tissue and peripheral blood: A dense fibrotic peri-tumoral stromal phenotype

**DOI:** 10.18632/oncotarget.24277

**Published:** 2018-01-19

**Authors:** Roshanak Derakhshandeh, Sonia Sanadhya, Kyu Lee Han, Haiyan Chen, Olga Goloubeva, Tonya J. Webb, Rania H. Younis

**Affiliations:** ^1^ Department of Oncology and Diagnostic Sciences, School of Dentistry, University of Maryland Baltimore, Baltimore, Maryland, USA; ^2^ Oral Pathology Consultants, School of Dentistry, University of Maryland Baltimore, Baltimore, Maryland, USA; ^3^ Department of Dental Public Health, School of Dentistry, University of Maryland Baltimore, Baltimore, Maryland, USA; ^4^ Department of oral Pathology, Faculty of Dentistry, Alexandria University, Alexandria, Egypt; ^5^ Department of Epidemiology and Public Health, School of Medicine, University of Maryland Baltimore, Baltimore, Maryland, USA; ^6^ Department of Microbiology and Immunology, School of Medicine, University of Maryland Baltimore, Baltimore, Maryland, USA; ^7^ The Marlene and Stewart Greenebaum Cancer Center, University of Maryland Baltimore, Baltimore, Maryland, USA

**Keywords:** semaphorin 4D, head and neck cancer, fibrotic stroma, non-inflamed, peripheral blood

## Abstract

The search for stromal biomarkers in carcinoma patients is a challenge in the field. Semaphorin 4D (Sema4D), known for its various developmental, physiological and pathological effects, plays a role in pro and anti-inflammatory responses. It is expressed in many epithelial tumors including head and neck squamous cell carcinoma (HNSCC). Recently, we found that HNSCC-associated Sema4D modulates an immune-suppressive, tumor-permissible environment by inducing the expansion of myeloid derived suppressor cells. The purpose of this study was to determine the value of Sema4D as a biomarker for the peri-tumoral stromal phenotype in human HNSCC. Our data showed Sema4D^+ve/high^ tumor cells in 34% of the studied cohort with positive correlation to Stage III (p=0.0001). Sema4D^+ve/high^ tumor cells correlated directly with dense fibrotic peri-tumoral stroma (p=0.0001) and inversely with infiltrate of Sema4D^+ve/high^ tumor-associated inflammatory cells (TAIs) (p=0.01). Most of the Sema4D^+ve/high^ TAIs were co-positive for the macrophage biomarker CD163. Knockdown of Sema4D in WSU-HN6 cells inhibited collagen production by fibroblasts, and decreased activated TGF-β1 levels in culture medium of HNSCC cell lines. In a stratification model of HNSCC using combined Sema4D and the programmed death ligand 1 (PDL-1), Sema4D^+ve/high^ tumor cells represented a phenotype distinct from the PDL-1 positive tumors. Finally,Sema4D was detected in plasma of HNC patients at significantly higher levels (115.44, ± 39.37) compared to healthy donors (38.60± 12.73) (p <0.0001). In conclusion, we present a novel HNSCC tumor stratification model, based on the expression of the biomarker Sema4D. This model opens new avenues to novel targeted therapeutic strategies.

## INTRODUCTION

Head and neck squamous cell carcinoma (HNSCC) is the sixth most common malignancy worldwide. In the US alone, 61,760 new cases and 13,190 deaths were expected to occur in the year 2016 [1]. The proximity to vital structures can significantly limit surgical approach and radiotherapy. Due to field cancerization, patients can suffer multiple primaries or recurrences with a 6 months average survival rate for advanced cases [2–4]. Tumor cell interaction within the microenvironment and their modulation of angiogenic factors, inflammation and fibrosis in the peri-tumoral stroma is essential for tumor progression [5–7]. Understanding the mechanisms that influence tumor inflammation and how to utilize the inflammatory mediators as readers that can predict the peri-tumoral stromal phenotype and accordingly patients’ differential response to several therapeutic modalities is essential [8, 9].

Semaphorin 4D (Sema4D) is a glycoprotein that belongs to group IV of the semaphorin family and can be present in a secreted or membrane bound form [10]. It was originally described as a repulsive axon guidance molecule in the developing nervous system [11]. Later on, its role in a broad range of physiological and pathological processes including angiogenesis, bone remodeling and regulation of the immune system was reported [12–15]. In the immune system, monocytes, neutrophils, B cells, and T cells can differentially express Sema4D; with expression being highest in activated T cells. Depending on the concentration of Sema4D and the cellular context, it can either play a pro-inflammatory role, co-activating antigen presenting cells, plasma cells and T cells [16, 17], or it can induce anti-inflammatory cytokine production from myeloid cells and inhibit their migration, conferring immune suppression [18–21].

Sema4D is over expressed in HNSCC, prostate, colon, breast, lung, cervical and ovarian malignancies [22–25] and it was described to induce tumor angiogenesis through binding to its high affinity receptor Plexin-B1 on endothelial cells promoting their proliferation and organization [24]. Also binding of Sema4D to its high affinity receptor Plexin-B1, was shown to mediate downstream activation of RhoA and subsequent microtubule organization enhancing cellular motility and tumor invasion, correlating with poor prognosis [24, 26–29].

Studies from our lab have shown that HNSCC-associated Sema4D leads to immune suppression through the induction of myeloid derived suppressor cells (MDSC) (CD33^+^, CD11b^+^, HLA-DR ^-/low^), with subsequent regulatory T cell (Treg) differentiation, and downregulation of T-helper cells (Th1) and cytotoxic T lymphocytes (CTLs) [21]. Sema4D expression by HNSCC, also resulted in a concomitant increase in signature suppressor cytokines production by MDSC, including TGF-β1 [21]. TGF-β1 is known for its immunosuppressive effects within the tumor microenvironment, and represents a major obstacle in cancer immunotherapy [30]. It inhibits anti-tumor immunity at the level of Th cells, CTLs, dendritic cells, macrophages, natural killer cells and B cells, while serving as a differentiation factor for Tregs [30, 31]. It is also known as the main fibroblast mitogen that induces cancer associated fibroblasts (CAFs) and tumor fibrosis [32–37].

The upregulation of the immune inhibitory molecule programmed death ligand 1 (PD-L1) is one of the mechanisms by which tumors escape immune surveillance. This was observed in several malignancies, as expressed by the tumor cells and the dendritic cell population of the tumor-associated inflammatory cells (TAIs) or by both. Several studies describe PD-L1 association with TAIs and poor prognosis [38–42]. The blockade of the PD-1 receptor or its ligand PD-L1 is one of the novel approaches for cancer immunotherapy that induced unprecedented objective response in up to 20- 30% of HNSCC patients [4, 39, 43, 44]. Yet, the response remains limited to certain patients that seem to show evidence of T-cell inflamed tumors, while the non-inflamed dense peri-tumoral stromal phenotype is resistant to several therapeutic modalities [9, 43, 45–50]. Further investigation of the association between several inflammatory mediators and the peri-tumoral stromal phenotype, can enhance our understanding of tumor stratification and hence response to therapy.

In this study, we investigated the immunohistochemical expression of Sema4D by HNSCC tumor cells in correlation to clinical parameters and the peri-tumoral stromal phenotype. The peri-tumoral phenotype was assessed in terms of fibrosis and TAIs infiltration, as well as PD-L1 expression. We showed that high levels of Sema4D expression (Sema4D^+ve/high^) by HNSCC tumor cells significantly correlated with clinical staging, being highest in stage III. Sema4D^+ve/high^ expression in tumor cells correlated significantly with dense fibrotic non-inflamed peri-tumoral stroma and inversely with infiltrating Sema4D^+ve/high^ TAIs. Interestingly, knockdown of Sema4D in a HNSCC cell line, showed inhibition of collagen production by fibroblasts and a decrease in activated TGF-β1 levels in culture medium of tumor cells. Sema4D^+ve/high^ tumor cells subtype was identified as a unique subset of HNSCC tumors distinct from the PD-L1^+ve/high^ tumor cells, allowing for tumor stratification based on differential expression of Sema4D and PD-L1. Furthermore, we detected high levels of circulating Sema4D, in HNSCC patients’ plasma compared to healthy donors. The data presented provide a novel role for Sema4D in the context of fibrosis and inflammation within the tumor microenvironment and demonstrate its value as a novel biomarker for HNSCC stromal phenotype.

## RESULTS

### Sema4D expression by tumor cells and clinical staging

Sema4D expression has been described in several epithelial malignancies, but to our knowledge, there are no studies investigating whether it correlates with clinical staging in HNSCC [29, 51]. We performed immunohistochemical (IHC) analysis on a tissue microarray (TMA) of 200 human tissue cores. These included 169 cases of Head and neck cancer (HNC), 6 normal adjacent to tumor and 25 cases of control-inflamed tissue. We excluded 6 control specimens and 16 HNSCC of overlapping cases or tissue lacking epithelial component. The TMA also included 9 primary salivary gland adenocarcinomas (SGA), with 2 control minor salivary gland tissues, four metastatic HNSCC to lymph nodes and 4 metastatic SGA to the lymph nodes. Statistical analysis was conducted only on the primary HNSCC (n=136) cases, for which 17 inflamed epithelial tissue served as control. The control consisted of 4 inflamed epiglottis tissues, 10 inflamed tongue tissues, 2 normal lingual mucous membrane tissues, and 1 inflamed laryngeal tissue. (Supplementary Figure 1A and 1B).

The normal oral epithelium was negative for Sema4D (Sema4D^-ve/low^) (Supplementary Figure 2A-2B). Reactive inflamed surface epithelium showed weak to moderate positivity of Sema4D. The scattered inflammatory cells in the inflamed tissue sections were also positive for Sema4D (Supplementary Figure 2C-2F). The normal tissue adjacent to tumor margin (NAT) was Sema4D positive to strongly positive (Sema4D^+ve/high^) in the basal and lower prickle cell (Supplementary Figure 2G-2H). While the normal salivary acini were negative for Sema4D, the salivary ductal epithelium was positive. Inflamed laryngeal epithelium as well as the infiltrating inflammatory cells were Sema4D positive (Supplementary Figure 3A, 3B and 3C).

**Figure 1:**
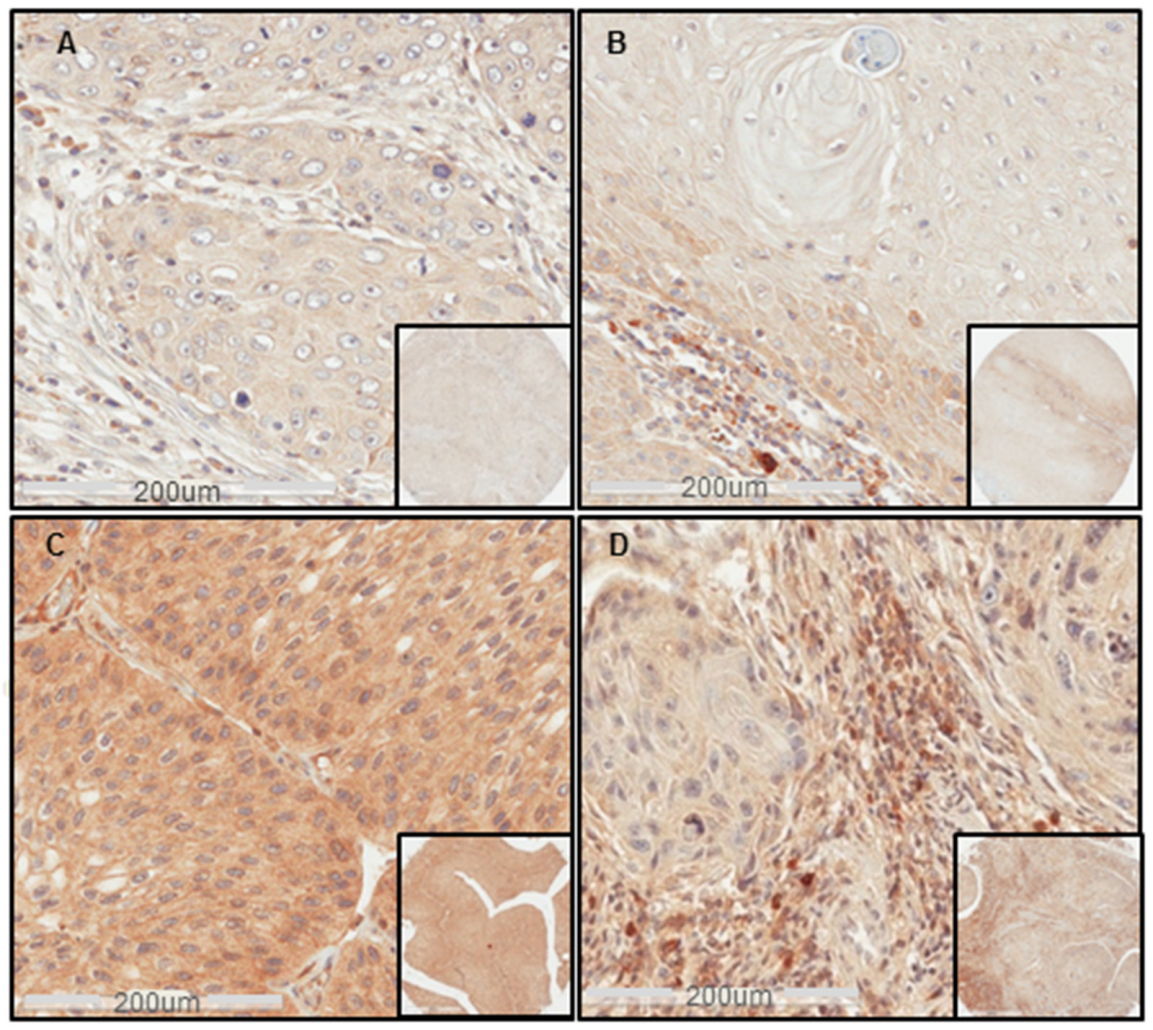
Sema4D expression in different clinical stages of HNSCC **(A)** IHC shows Sema4D^-ve/low^ expression in tumor cells of a stage I SCC of the nasal cavity. **(B)** Sema4D^-ve/low^ expression in tumor cells of Stage II SCC of the larynx. Moderate stromal infiltration with Sema4D^+ve/high^ TAIs. **(C)** Sema4D^+ve/high^ expression in tumor cells of a stage III SCC of the upper Jaw. **(D)** Sema4D^-ve/low^ expression in tumor cells of stage IV SCC of the gingiva with the peri-tumoral stroma showing heavy infiltration of Sema4D^+ve/high^ TAIs. (20x; 200um scale, inset: 40x).

Statistical analysis showed no significant difference between HNSCC and the inflamed control tissue (Supplementary Table 1). In HNSCC, Sema4D^+ve/high^ expression in tumor cells correlated significantly with stage III HNC (p<0.0001) (Table 1). Tumor cells were generally Sema4D^-ve/low^ in stage I and II, Sema4D^+ve/high^ in stage III and Sema4D^-ve/low^ in stage IV (Figure 1A–1D). Only one out of four metastatic SCC to lymph nodes had Sema4D^+ve/high^ expression in the tumor cells, while the others were Sema4D^-ve/low^. A statistically insignificant positive association between Sema4D expression in primary tumor cells and nodal metastasis was observed, specifically patients with nodal metastasis had higher levels of Sema4D staining in the primary tumor (p= 0.12) (Table 1) (Supplementary Figure 3D-3F). A positive correlation between Sema4D^+ve/high^ tumor cells and the pharyngeal location of HNSCC was detected (p=0.03) (Supplementary Table 2). There was no association detected between the Sema4D expression in tumor cells and other demographic variables, nor the histological grade of HNSCC (Supplementary Table 2). Out of the limited number of SGA, Sema4D^+ve/high^ expression was observed in 7 (77%) out of the 9 primary (Supplementary Figure 3C) and 3 out of 4 metastatic SGA to lymph nodes. The cut off value to consider Sema4D^+ve/high^ in tumor cells was strong diffuse positivity, that translated as 5x10^5^ strong pixel intensity guided by the standardized digital analysis (Supplementary Figure 4).

**Table 1:** Sema4D expression in HNSCC tumor cells in correlation to clinical staging, stromal fibrosis and Sema4D^+ve/high^ TAIs

	Sema4D expression in tumor cells		Total	P-value
	-ve / low No (%)	+ve / high No (%)		
**Tumor stage**				
I+II	47(80)	12 (20)	59 (43)	0.0001
III	16 (40)	24 (60)	40 (29)	
IV	27(73)	10 (27)	37 (27)	
Total	90 (66)	46 (34)	136 (100)	
**Lymph nodal metastasis**				
No	70 (70)	30 (30)	100 (74)	0.12
Yes	20 (56)	16 (44)	36 (26)	
Total	90 (66)	46 (34)	136 (100)	
**Stromal Fibrosis**				
Delicate/moderate	62 (82)	14 (18)	76 (70)	<0.0001
Dense	8 (24)	25 (76)	33 (30)	
Total	70 (64)	39 (36)	109 (100)	
**Sema4D^+ve/high^TAIs infiltrate**				
No/low	26 (53)	23 (47)	49 (37)	0.011
Yes/high	62 (75)	21 (25)	83 (63)	
Total	88 (67)	44 (33)	132 (100)	

### Sema4D expression by tumor cells in correlation to the peri-tumoral stromal phenotype

**Figure 2:**
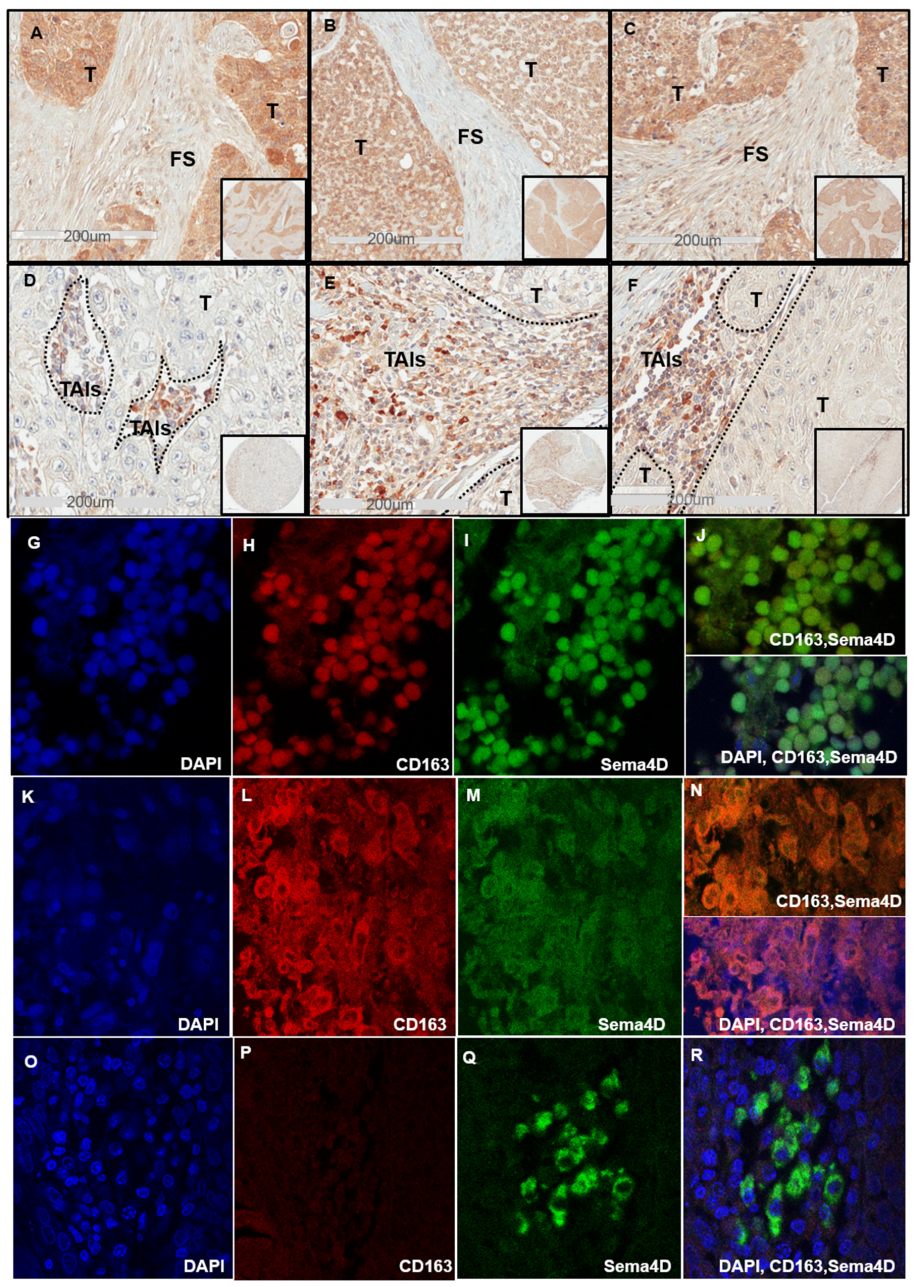
Sema4D^+ve/high^ tumor cells correlates directly with dense peri-tumoral stroma and inversely with stromal Sema4D^+ve/high^ TAIs **(A)** IHC shows Sema4D^+ve/high^ expression in tumor islands with surrounding dense fibrotic stroma in a stage I SCC of the maxillary sinus. **(B)** Sema4D^+ve/high^ expression in tumor islands of a stage II Adenoid Cystic Carcinoma of minor salivary glands arising in the tongue, associated with dense fibrotic stroma. **(C)** Sema4D^+ve/high^ expression in stage III SCC of the larynx with surrounding dense fibrotic stroma. **(D)** Stage III laryngeal SCC with Sema4D^+ve/high^ TAIs and Sema4D^-ve/low^ tumor cells. **(E)** Sema4D^+ve/high^ TAIs and Sema4D^-ve/low^ tumor cells of stage IV SCC of the larynx. **(F)** Sema4D^+ve/high^ expression in TAIs corresponding to Sema4D^-ve/low^ in tumor cells of stage IV SCC of gingiva. **(G-J)** IF of stage II nasal HNSCC with TAIs showing small monoyctic features with scant cytoplasm, (G) DAPI positive monocytes, (H) CD163 positive monocytes, (I)Sema4D positive monocytes, (j) merge of Sema4D and CD163 in monocytes (upper panel) and with DAPI(lower panel). **(K-N)** IF of Stage II nasal SCC with TAIs showing large histiocytic features with abundant cytoplasm, (K) DAPI positive histiocytes, (L) CD163 positive histiocytes, (M) Sema4D positive histiocytes, (N) merge of Sema4D and CD163 (upper panel) with DAPI (lower panel). **(O-R)** IF of Stage II SCC of the tongue with TAIs showing plasmacytoid/monocytoid features, (O) DAPI, (P) CD163 negative monocytoid cells (Q) Sema4D positive monocytoid cells, (R) merge Sema4D, CD163 and DAPI. T; Tumor, FS; fibrotic stroma, TAIs; tumor associated inflammatory cells, (IHC 20x; 200um scale, in set: 40x), (IF, oil immersion at 100x).

The peri-tumoral stromal phenotype is a very important factor in reflecting the tumor complexity [6, 7]. To determine whether Sema4D has a role in modulating the peri-tumoral stroma, we examined the tumor microenvironment in all the provided specimens in terms of inflammation and stromal density. Sema4D^+ve/high^ expression by tumor cells of primary HNSCC correlated directly with a dense fibrotic peri-tumoral stroma, representing 23% of the total cohort (Figure 2A-2C) (p<0.0001) (Table 1). The data also showed 76% of the cases with dense peri-tumoral stroma to be Sema4D^+ve/high^ in tumor cells (Table 1).

We further investigated the peri-tumoral stroma for the extent of inflammation. We noticed that when considerable TAIs were present, almost all showed positivity for Sema4D (Sema4D^+ve/high^ TAIs). The stroma in Stage I and II HNSCC showed mild to moderate infiltrate of Sema4D^+ve/high^ TAIs (Figure 1A and 1B) (Supplementary Figure 5). Stage III HNSCC tumors were generally less inflamed, to more dense fibrotic (Figure 2A-2D). Stage IV tumors showed moderate Sema4D^+ve/high^ TAIs infiltration in most of the specimens (Figure 2E-2F). Sema4D^+ve/high^ TAIs proved to be statistically negatively associated with Sema4D expression in tumor cells (p= 0.011), where Sema4D^-ve/low^ expression in tumor cells correlated with increased infiltration of Sema4D^+ve/high^ TAIs in the peri-tumoral stroma (Table 1). No correlation between Sema4D^+ve/high^ TAIs and other patients’ demographics, histological grading or clinical staging was detected (Supplementary Table 3).

Most of the Sema4D^+ve/high^ TAIs observed were small and monocytic with scant cytoplasm, while few had plasmacytoid features of eccentric nucleus with abundant cytoplasm and very strong Sema4D expression (Figure 2E-2F). We wanted to investigate if the Sema4D^+ve/high^ TAIs observed in the current cohort of HNSCC are of the tumor associated macrophage lineage. We co-stained one TMA of 30 HNSCC, with Sema4D and the monocytic/macrophage marker CD163 using immunofluorescence (IF). Indeed, several areas showed evidence of Sema4D and CD163 co-staining, suggestive of the macrophage lineage of most of the Sema4D^+ve^ TAIs (Figure 2G-2N). We also observed TAIs with monocytoid features that was strongly positive to Sema4D, yet negative for CD163 (Figure 2O-2R).

### Sema4D/Plexin-B1/TGF-β1 pathway plays a role in modulating the fibrotic peri-tumoral stromal phenotype

**Figure 3:**
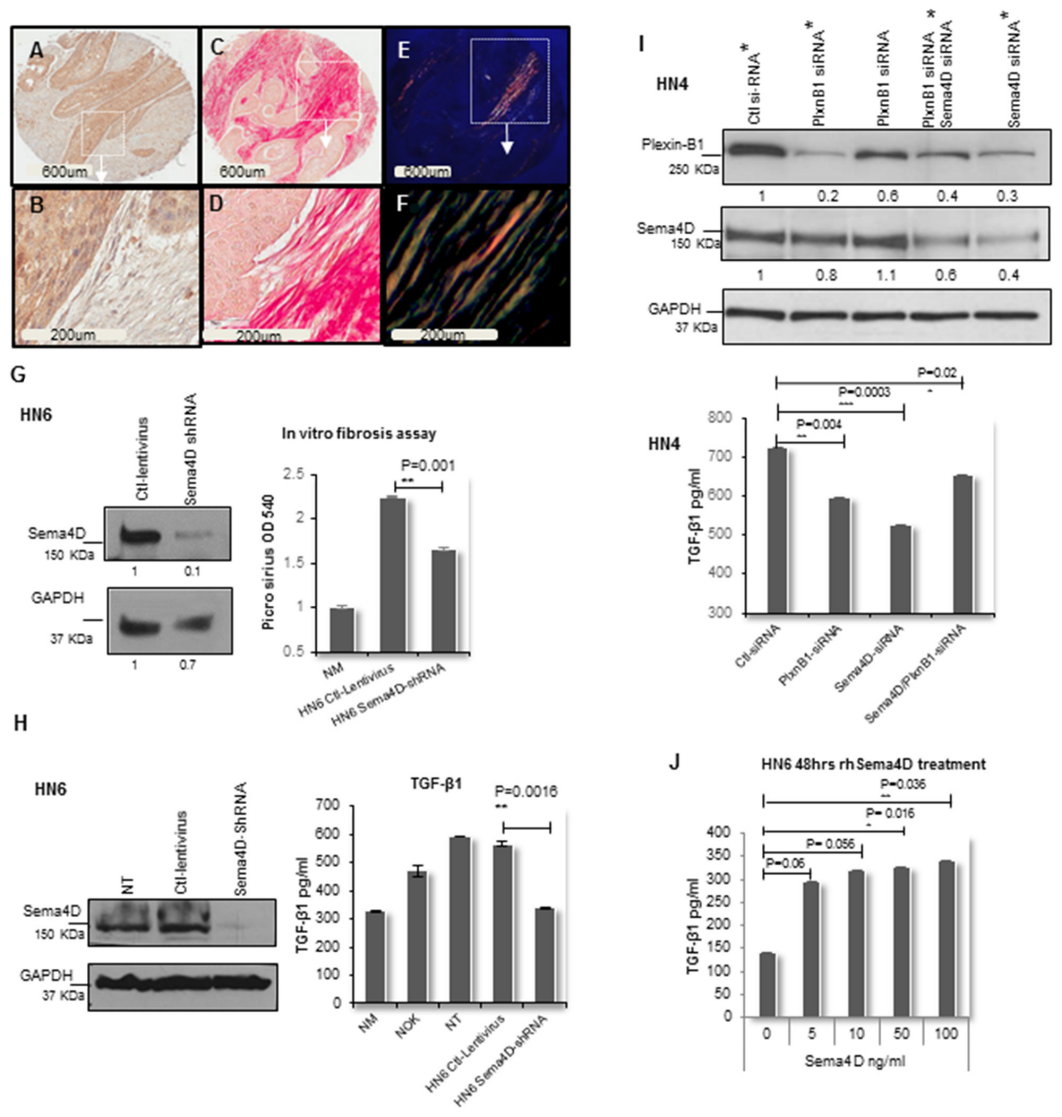
Sema4D-Plexin B1 pathway plays a role in TGF-β1 production by tumor cells **(A)** IHC of stage III laryngeal SCC showing Sema4D^+ve/high^ tumor cells (2x). **(B)** The peri-tumoral stroma is dense fibrotic and non-inflamed (20x). **(C)** PSR stain examined in bright field microscopy (2x). **(D)** The PSR stains the dense peri-tumoral fibrotic stroma red (20x). **(E)** PSR stain examined under polarized light (2x). **(F)** Orange red to yellow green birefringence specific to collagen I and III respectively, is observed in the peri-tumoral stromal (20x). **(G)** *In vitro* fibrosis assay. Graph illustrates decreased collagen production by fibroblasts when incubated in HN6 tumor conditioned media of Sema4D-shRNA for 72hrs compared to ctl-lentivirus. PSR stained the extra cellular collagen and was estimated using OD. Immunoblot illustrates the extent of inhibition of Sema4D using shRNA. **(H)** Downregulation of TGF-β1 production by HN6 cells upon Sema4D-shRNA, compared to transfection control, NT and NOK. TGF-β1 detected using ELISA. Immunoblot shows the extent of Sema4D inhibition. **(I)** Upper panel shows immunoblot of siRNA silencing of Plexin-B1, Sema4D and combined Plexin-B1/Sema4D, which reflected with a decrease in the activated TGF-β1 level in HN4 CM as detected by ELISA (lower panel). The immunoblot shows 2 titrations of Plexin-B1 siRNA. The CM tested for TGF-β1 was obtained from the siRNA titrations labeled with asterisks. **(J)** TGF-β1 upregulation in CM upon treatment of HN6 tumor cells with rhSema4D for 48hrs. CM; culture medium, NT; non-transfected, NOK; normal oral keratinocytes and NM; Normal media, PSR; Picro sirus red stain, rhSema4D; recombinant human Sema4D.

To further investigate the Sema4D^+ve/high^ HNSCC tumor cells with dense peri-tumoral stromal phenotype (Figure 2A-2C), we stained one TMA of 60 HNSCC for collagen. The Picro Sirius Red (PSR) stain, for which polarization (birefringence) is specific for collagen was used [52]. PSR stained bright red the dense peri-tumoral stroma associated with the Sema4D^+ve/high^HNSCC tumor cells (Figure 3A-3D). Under polarized light, PSR illustrated strong orange red and yellowish green birefringence characteristic of the thick collagen I and collagen III fibers, respectively (Figure 3E-3F).

To investigate the direct role of Sema4D in collagen production, we carried out an *in vitro* fibrosis assay [53]. We generated stable Sema4D knockdown and controls using the HN6 cell line, derived from the base of the tongue [54]. Fibroblasts were cultured in conditioned medium from the HN6 cells with Sema4D-shRNA. Production of extracellular collagen by the fibroblasts was significantly decreased in the Sema4D knockdown group compared to control, as indicated by the PSR staining of extracellular collagen (Figure 3G).

We previously showed that inhibition of Sema4D in HNC cell lines can downregulate production of the master of fibrosis; TGF-β1, by myeloid cells [55, 56] [21]. To further investigate whether Sema4D can directly induce production of TGF-β1 by HNSCC cells and conversely if inhibition of Sema4D would affect the production of TGF-β1 by tumor cells, we used the HN6 stable Sema4D knockdown and controls [54]. Activated TGF-β1 was detected in high levels in the culture medium (CM) of HN6 cells, compared to control normal oral keratinocytes (NOK). Inhibition of Sema4D in HN6 cells using shRNA, significantly reduced the level of activated TGF-β1 in the CM of the tumor cells to levels comparable to NOK and to that present in normal cell culture medium (Figure 3H).

Plexin-B1 functions as a high affinity receptor for Sema4D on epithelial cells [26, 57]. To investigate if Sema4D mediates TGF-β1 production, through binding to its receptor Plexin-B1 on tumor cells, we used siRNA system for transient silencing of Plexin-B1 in several HNSCC cell lines of the oral tongue. Plexin-B1 silencing showed significant reduction of activated TGF-β1 levels in CM of HN4 (Figure 3I), SCC9, HN13 and HN6 (Supplementary Figure 6A-6D). Combined inhibition of Sema4D and Plexin-B1 showed almost the same percentage of TGF-β1 reduction upon inhibition of either Plexin-β1 or Sema4D alone. A reduction in Plexin B1 expression upon Sema4D silencing was observed (Figure 3I).

In addition, we treated HN6 cells with recombinant human Sema4D (rhSema4D) to check for a direct increase in TGF-β1. Evidence of increased levels of activated TGF-β1 in the CM were detected with low doses of Sema4D (Figure 3J). To check for the activation of the Sema4D/PlexinB1 pathway, we blotted for phospho Erk as a downstream activation marker. Low doses of rhSema4D treatment for 20 minutes [57], showed evidence of increased Erk phosphorylation (Supplementary Figure 6E) and increased level of activated TGF-β1 in tumor CM up to 72hrs (Supplementary Figure 6F).

### HNSCC stratification according to Sema4D and PD-L1 expression

**Figure 4:**
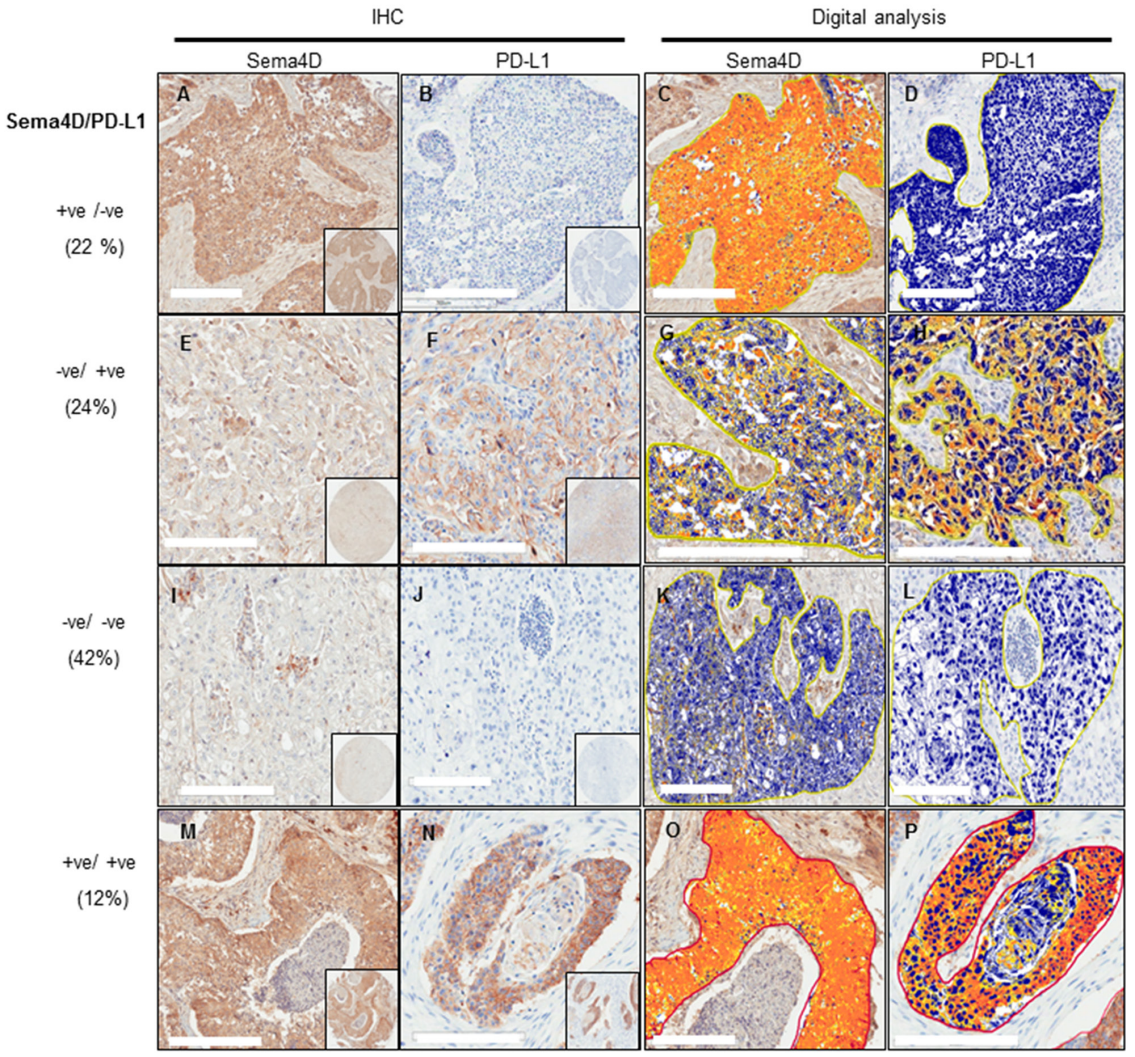
Stratification of HNSCC tumors into 4 subtypes according to Sema4D and PD-L1 expression **(A**-**D)** Sema4D^+ve/high^/PD-L1^-ve/low^ tumor subtype. (A) A stage III SCC of the larynx with Sema4D^+ve/high^ tumor cells surrounded with fibrotic peri-tumoral stroma. (B) The same tumor as in (A) showed PD-L1^-ve/low^ expression (C) Total strong pixel intensity for Sema4D stain in (A) ∼ 2x10^6^. (D) Total strong pixel intensity for PD-L1 stain in (B) <10^4^. **(E-H)** Sema4D^-ve/low^/PD-L1^+ve/high^ tumor subtype. (E) A stage IV SCC of the lip showed Sema4D^-ve/low^ tumor cells with Sema4D^+ve/high^ TAIs in the peri-tumoral stroma. (F) Same tumor as in (E) showed PD-L1^+ve/high^ tumor cells, TAIs were PD-L1^–ve/low^. (G) Total strong pixel intensity of Sema4D stain in (E) of ∼ 1.5x10^5^. (H) Total strong pixel intensity for PD-L1 stain in (F) ∼ 5.3x10^6^. **(I-L)** Sema4D^-ve/low^/PD-L1^-ve/low^ tumor subtype. (I) Tumor cells of a stage III SCC of the larynx were Sema4D^-ve/low^, TAIs were Sema4D^+ve/high^. (J) The same tumor as in (I), PD-L1-^ve/low^ for tumor cells and TAIs. (K) Total strong pixel intensity for tumor stain in (I) <10^4^. (L) Total strong pixel intensity for PD-L1 (J) ∼10^4^. **(M-P)** Sema4D^+ve/high^ /PD-L1^+ve/high^ tumor subtype. (M) A stage III SCC of the larynx, with tumor cells Sema4D^+ve/high^ with peri-tumoral fibrotic stroma showing focal Sema4D^+ve/high^ TAIs. (N) PD-L1^+ve/high^ in same tumor as in (M) TAIs were PD-L1 negative. (O) Total strong pixel intensity for Sema4D stain in (M) 1.5x10^6^. (P) Total strong pixel intensity for PD-L1 stain in (N) ∼ 4 x10^6^. (20x; 200um scale, inset: 40x,pixel intensity was calculated as the mean of 3 annotation layers per tumor core taken at 20x; at area 0.2mm^2^. Cut off value for positive is 5x10^5^).

Programmed death-ligand 1 (PD-L1) expression has been linked to poor prognosis [39, 58, 59]. The inhibition of PD-1/PD-L1 pathway has shown promising overall response rates in many clinical studies. Yet patients’ response had been linked to the initial presence of an inflamed tumor microenvironment [38, 60–62]. Our findings that tumor cells expressing Sema4D^+ve/high^ correlated positively with a non-inflamed dense fibrotic stroma and inversely with Sema4D^+ve/high^ TAIs, triggered the question; if HNSCC tumors expressing high Sema4D represent a distinct tumor phenotype other than the inflamed, PD-L1 positive tumors. To investigate whether Sema4D and PDL-1 were differentially expressed within tumor tissue, we assessed for PD-L1 expression in the same HNSCC tumor set studied for Sema4D. The specimens from the normal epithelium, normal salivary glands and epiglottis, were negative for PD-L1 as well as the normal epithelium from the tumor margin (Supplementary Figure 7A-7B) (Supplementary Table 4). When present PD-L1 expression in tumor cells, was mainly membranous, and often with strong positive cytoplasmic staining. Total PD-L1^+ve/high^ in tumor cells and TAIs were observed in 36% of 135 primary malignancies compared to 34% Sema4D^+ve/high^ tumor cells. Sema4D^+ve^/PD-L1^–ve^ tumors accounted for 22%, while Sema4D^–ve^/PD-L1^+ve^ tumors were 24% out of total (Figure 4) (Supplementary Figure 7C-7D) (Table 2). PD-L1 expression in tumor cells did not correlate with tumor staging (p=0.58) but showed a positive correlation with lymph node metastasis (p=0.016) (Table 3) (Supplementary Table 5). PD-L1 showed a statistically insignificant inverse correlation with peri-tumoral stromal fibrosis (p=0.14), while PD-L1 correlated directly with Sema4D^+ve/high^ TAIs (p=0.007) (Table 3). Only 16 cases (12%) out of the total cohort were Sema4D/ PDL-1 double positive. The double positive cases showed Sema4D^+ve/high^ TAIs. Fifty percent of the PD-L1/Sema4D double negative cases showed Sema4D^+ve/high^ TAIs (Figure 4, Table 2).

**Table 2:** Differential expression of Sema4D and PD-L1 in HNSCC

	Sema4D in tumor cells		Total	P value
	-ve / low No (%)^*^	+ve / high No (%)^*^		
**PD-L1 in tumor cells**				
-ve/low	65 (66)	34 (34)	99 (73)	0.91
+ve/high	24 (67)	12 (33)	36 (27)	
Total	89 (66)	46 (34)	135 (100)	
**PD-L1 in tumor cells and/or TAIs**				
-ve/low	56 (65)	30 (35)	86 (64)	0.79
+ve/high	33 (67)	16 (33)	49 (36)	
Total	89 (66)	46 (34)	135 (100)	

**Table 3:** PD-L1 expression in relation to tumor staging, stromal fibrosis and Sema4D expressing TAIs

	Total PD-L1 expression in tumor cells and TAIs		Total	P-value
	-ve/low No (%)	+ve/high No (%)		
**Tumor stage**				
I+II	38 (66)	20 (34)	58 (43)	0.58
III	26 (65)	14 (35)	40 (30)	
IV	22 (59)	15 (41)	37 (27)	
Total	86 (64)	49 (36)	135 (100)	
**Nodal metastases**				
Yes	17 (47)	19 (53)	36 (27)	0.016
No	69 (70)	30 (30)	99 (73)	
Total	86 (64)	49 (36)	135 (100)	
**Stromal Fibrosis**				
Delicate/moderate	43 (57)	32 (43)	75 (69)	0.14
Dense	24 (73)	9 (27)	33 (31)	
Total	67 (62)	41 (38)	108 (100)	
**Sema4D^+ve/high^** **TAIs infiltrate**				
No	29 (80)	7 (20)	36 (34)	0.007
Yes	38 (54)	33 (46)	71 (66)	
Total	67 (63)	40 (37)	107 (100)	

### Sema4D detection in plasma of HNSCC patients

**Figure 5:**
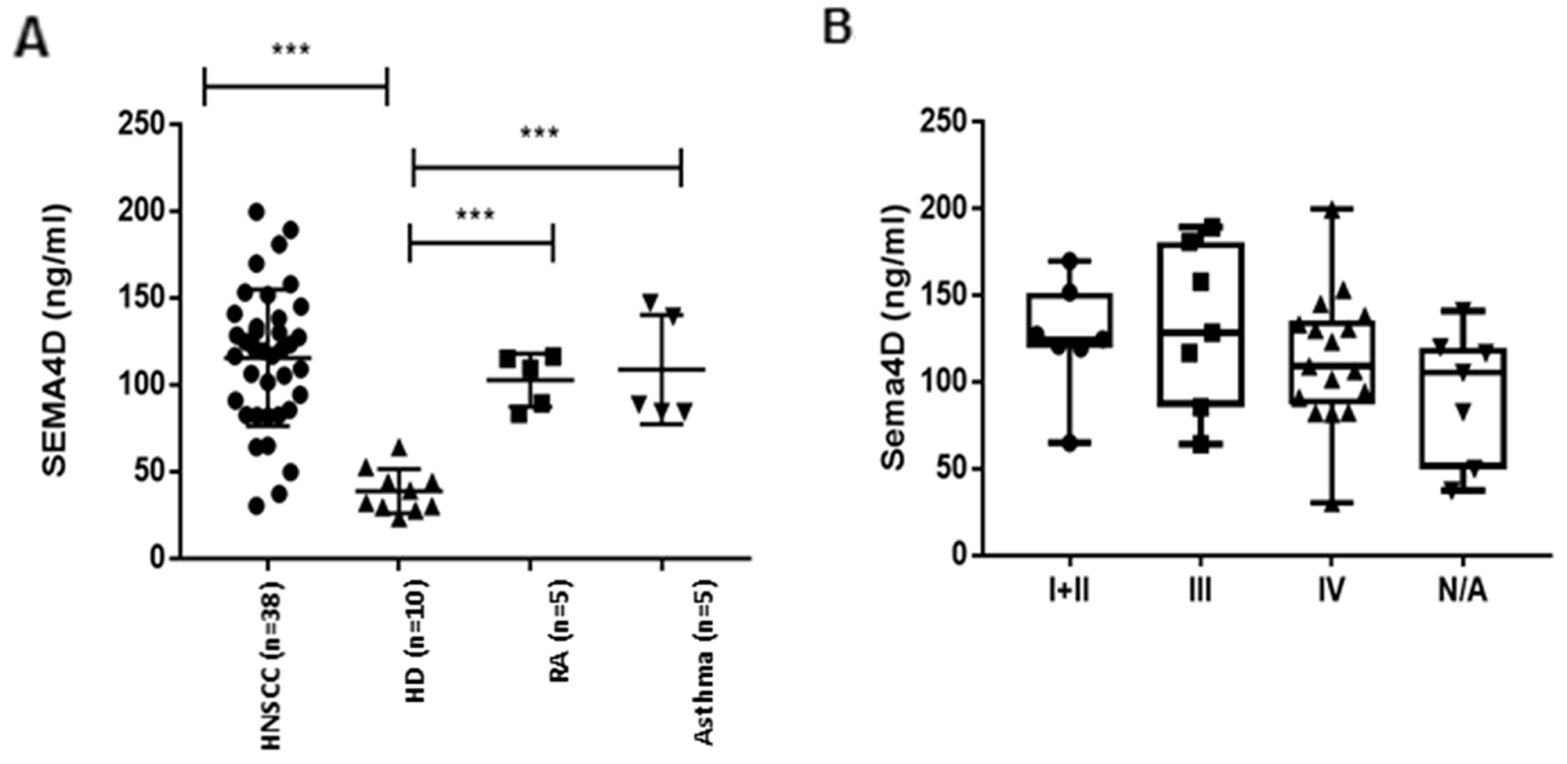
Analysis of Sema4D in plasma of HNC patients **(A)** ELISA assay of Sema4D in plasma of HNSCC patients compared to HD (p<0.0001), RA and Asthma patients. **(B)** Sema4D level in several stages of HNSCC. HD; Healthy donors, HNSCC; Head and Neck squamous cell carcinoma, RA; Rheumatoid arthritis, ATH; Asthma patients, N/A; stage information not available.

The current findings of Sema4D expression in tumor cells and in TAIs, in addition to our previous *in vitro* studies showing that HNSCC can produce soluble Sema4D in the tumor CM [21], raised the question of whether soluble Sema4D can be detected in the peripheral blood of HNSCC patients. ELISA was used to assess Sema4D levels in plasma of 10 healthy donors (HD) and 38 HNSCC patients. In the 10 HD, plasma Sema4D levels were 38.60± 12.73ng/mL, n=10. There was a statistically insignificant gender difference. The levels of plasma Sema4D in healthy donor males (33.5±11.2ng/mL, n = 5) were lower than that in females (43.7±13.15, n=5) (p=0.22). The demographics of the HNSCC patients in this study are summarized in Table 4. The levels of Sema4D in the HNSCC plasma was 115.44 ± 39.37ng/mL, n=38, that was significantly higher than those in HD (38.6± 12.73) (p<0.0001). No significant difference in plasma levels of Sema4D was detected between tumor stages (Figure 5) (Table 4). To test whether the higher levels of Sema4D in plasma are specific to the HNSCC cases, two different inflammatory diseases as controls, asthma patients and rheumatoid arthritis. We found no significant difference between the level of Sema4D in these two inflammatory conditions and HNSCC (Table 4) (Figure 5A). No correlation was detected between Sema4D plasma levels and other clinical or demographic parameters (Table 4).

**Table 4:** Sema4D analysis in plasma of HNC patients in relation to demographics and clinical characteristics

Characteristics	Number of patients	Sema4D (ng/ml) Mean, STD	P-value
**Mean age, STD (range)**	51.68,15.76 (18-86)	99.75, 45.26	0.07
**Race**			
White	37	110.28, 44.13	0.2
others	9	100.09, 59.51	
**Gender**			
Male	34	99.16, 42.46	0.15
Female	14	100.09, 59.51	
**Asthma (AS)**			
HNSCC	38	115.44, 39.37	0.72
AS	5	108.83, 31.55	
**Rheumatoid Arthritis (RA)**			
HNSCC	38	115.44, 39.37	0.31
RA	5	102.78, 15.29	
**Healthy donors (HD)**			
HNSCC	38	115.44, 39.37	<0.0001
HD	10	38.60, 12.73	
**Stage**			
I+II	7	125.58, 32.60	0.69
III+IV	24	122.77, 42.41	
**Location**			
Oral	14	121.74, 33.40	0.49
Pharynx, Larynx	23	111.36, 43.63	
**LN metastases**			
No	9	131.42, 38.35	0.3
Yes	21	115.75, 39.35	

## DISCUSSION

The extent and type of inflammation and the density of the stroma are two major determinants of the peri-tumoral stromal phenotype [5, 7, 63]. The availability of biomarkers that can monitor and read the tumor stroma and accordingly dictate how patients respond to treatment, is a gap that still needs to be investigated [64]. The current study presents Sema4D expressed by HNSCC tumor cells, as a biomarker for dense fibrotic peri-tumoral stroma, and presents a novel role for Sema4D in modulating the tumor microenvironment through upregulation of ECM collagen production by fibroblasts, and TGF-β1 by tumor cells.

Two main models have been described in literature for the peri-tumoral stromal phenotype. [8, 65]. One is a T cell inflamed subtype, in which the tumor associated T cells are functionally inhibited through anergy and T cell checkpoints. This subtype is likely to respond to check point inhibitors [66, 67]. The other subtype describes non-T cell infiltrated tumors with two main characteristics; a denser stroma and myeloid or macrophage inflammatory cells. This dense stroma acts as a barrier to T cell infiltration and confers an immune-privileged environment that hinders effective pre-existing immune responses, as well as adoptive immunotherapy from reaching the tumor. Hence, rendering this subtype resistant to immunotherapy compared to the inflamed one [65, 68]. Interestingly, we recently reported data supportive of Sema4D induction of MDSCs [21]. The two tumor subtypes observed in this study, according to Sema4D distribution: Sema4D^+ve/high^. HNSCC tumor cells with dense non inflamed stroma versus the Sema4D^-ve/low^ tumor cells with Sema4D^+ve/high^ TAIs, are reminiscent of the previously described non T-cell inflamed, versus the inflamed subtype, respectively [9, 65].

TGF-β1 acts on fibroblasts to induce fibrosis, and can suppress the immune response by inhibiting the functions of effector cells and increasing the numbers of Tregs and immature dendritic cells. It can also induce CAFs [36, 69–71], which can present with characteristics of MDSC, an additional feature of the non-inflamed tumor phenotype [72]. Our data shows a novel mechanism of TGF-β1 regulation downstream of Sema4D and its receptor Plexin-B1, in HNSCC cell lines (Figure 3G-3I) (Supplementary Figure 6A-6D). Several groups have shown that Sema4D binding to its receptor Plexin-B 1 can induce downstream activation of the RhoA/AKT pathway, as well as MAPK/ERK activation [26]. Interestingly, TGF-β1 translation requires RhoA/GTPase AKT activation, while MAPK/ERK can be involved in both TGF-β1 translation and transcription (Supplementary Figure 6E-F) [73]. Further investigation of how Sema4D/Plexin-B1 affect downstream translation and transcription of TGF-β1 is currently ongoing in our lab.

**Figure 6:**
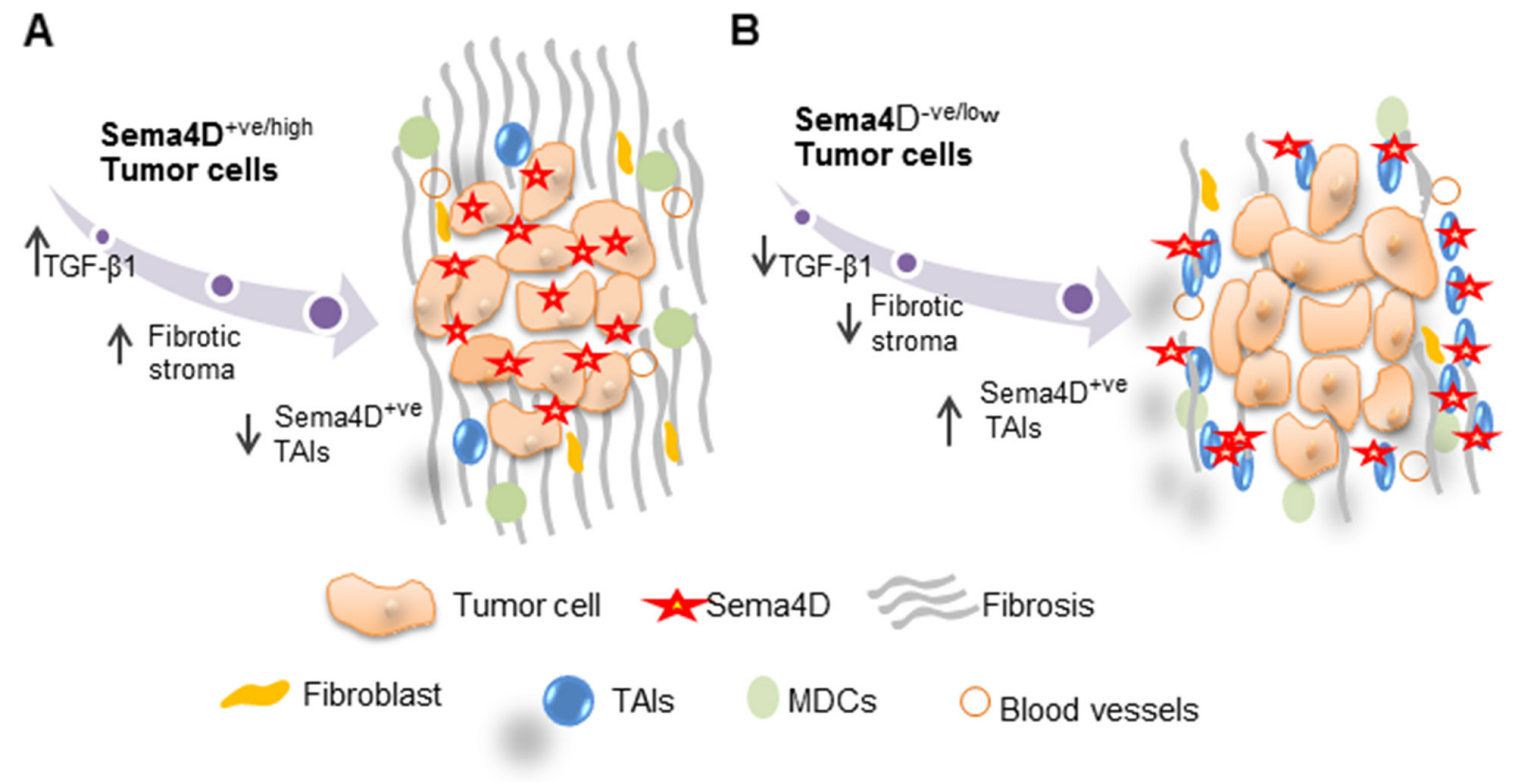
Model for Sema4D^+ve/high^ tumor cells with a fibrotic peri-tumoral stromal phenotype versus Sema4D^-ve/low^ tumor cells with Sema4D^+ve/high^ TAIs **(A)** Sema4D^+ve/high^ tumor cells induce increased production of TGF-β1 by tumor cells and collagen deposition by fibroblasts in the ECM. **(B)** Sema4D^-ve/low^ tumor cells with inflamed stroma. Sema4D^-ve/low^ tumors has decreased level of TGF-β1 production, less fibrotic and more into the loose peri-tumoral stroma with increased infiltrate of Sema4D^+ve/high^ TAIs. TAIs: Tumor Associated Inflammatory cells.

Collagen deposition accompanies tumor progression and its organization facilitates invasion. Collagen is readily identified during routine microscopic examination, and PSR staining allows imaging of the collagen structure especially when using polarized light [68]. In addition to fibroblasts, macrophages also can regulate collagen production [68]. The dense fibrotic peri-tumoral stroma observed in the current work, demonstrating collagen I and III, with Sema4D^+ve/high^ HNSCC tumor cells, in addition to the role for Sema4D in induction of collagen deposition by fibroblasts (Figure 3A–3G) (Figure 6), reveals another prospective by which Sema4D induces immune suppression. Matrix architecture was reported to define migration of T cells into the stroma of human lung tumor [69, 74] [68]. The dense fibrotic stromal phenotype rich with CAFs and collagen, specially type I, has long been linked with aggressive fatal malignancies and poor prognosis including pancreatic, lung, breast and HNSCC malignancies [68, 69, 74–79].

Whether the Sema4D^+ve/high^ TAIs subtype is an early or late event in the progression of the disease, or if the inflammatory profile of Sema4D^+ve/high^ TAIs is different between several stages of the tumor is yet to be determined. Also, if Sema4D^+ve/high^ TAIs provide a positive feedback in stage IV malignancy in which the tumor cells are Sema4D^-ve/low^ is not clear at this point. We observed that Sema4D is positive in cells bearing the monocytic/macrophage marker CD163 [80] [25], indicating that Sema4D is positive in the macrophage lineage of the tumor microenvironment. This might indicate a positive feedback mechanism, by tumor associated macrophages expressing Sema4D and might explain the two subtypes of Sema4D^+ve/high^ tumor cells versus Sema4D^+ve/high^ TAIs. Further phenotyping of the TAIs population in correlation to tumor staging and in correlation to Sema4D expression by tumor cells, will be important to confirm the suppressor cell phenotype versus other monocytic or lymphocytic cells. Specially that activated T cells are reported to express high levels of Sema4D and some of the Sema4D^+ve/high^ TAIs in the current specimens were negative for the CD163 macrophage biomarker (Figure 2) [12]. Interestingly, in the limited number of SGA samples examined, Sema4D^+ve/high^ tumor cells with non-inflamed stroma predominated. This suggests a role for Sema4D in modulating the stroma of other non-squamous tumors of the head and neck. Analysis of Sema4D and PD-L1 in relation to the stromal and inflammatory profile, in a larger sample of SGA would be informative.

Herein, we also present evidence showing that Sema4D^+ve/high^ tumor cells correlate significantly with stage III HNSCC. At early stages, Sema4D expression in tumor cells was low, (Figure 1A–1C) (Table 1), suggesting that Sema4D may confer an equilibrium state at stage III that can then develop into eventual escape in stage IV [81]. This is consistent with the role TGF-β1 plays in promoting tumor progression in the late stages of the disease through stimulation of tissue fibrosis, immune suppression, angiogenesis, enabling migration, invasion, epithelial mesenchymal transition and stem cell maintenance [82, 83]. Tumors also develop through several heterogeneous steps. The same type of malignancy might be heterogeneous in the inflammatory profile and in antigenic burden from one stage to the other [84]. Whether Sema4D expression by tumor cells correlates with mutational burden in stage III malignancy versus stage IV, is still to be investigated. Sema4D increased expression in epithelial malignancy has been described to be downstream of the HIF1α transcription factor under hypoxic conditions [85]. The fact that in stage III, epithelial tumor islands can reach a substantially bigger size with more hypoxic effect is to be considered. We did notice large epithelial islands in some, but not all of the Sema4D^+ve/high^ tumors. It is worth mentioning that a limitation of the current study is that it included only stage IV malignancy with loco-regional metastases and did not include stage IV with distant metastases. Sema4D expression in such stages is yet to be investigated. In addition, no information regarding smoking history or HPV status was available, leaving our findings more descriptive rather than conclusive. All reported p-values are nominal, the adjustments for multiple testing was not done. The p-values reported that were of marginal statistical significance still may be indicative of some trends. We will need to explore our research hypothesis further.

Significant advances in the field of cancer immunotherapy have been achieved. However, there is a substantial number of patients who do not respond and patient selection remains a pre-requisite [86, 87]. Combined analysis of the tumor cells and the peri-stromal phenotype is a promising strategy for tumor stratification and personalized treatment [48]. In the current cohort, PD-L1^+ve/high^ tumor cells were 24 (18%) cases, while combined PD-L1^+ve/high^ tumor cells and TAIs were 33 (24%) cases, out of the studied HNSCC (Table 2). Interestingly, in clinical trials of previously treated patients with recurrent SCC of the head and neck, the PD-1 inhibitor showed an overall response rate of ∼20%-30% [43, 44, 50]. Our findings that Sema4D^+ve/high^ represented 46 cases (34%) of the current HNSCC cohort, out of which 30 cases (65%) were PD-L1^-ve/low^, indicates that Sema4D^+ve/high^ tumors, represent a significant subgroup of HNC that might be responsive to Sema4D inhibition as a monotherapy, specifically and PDL-1 negative tumors correlated with poor prognosis in certain stage III malignancies [84]. Sema4D can also be targeted, in combination therapy with checkpoint inhibitors, since 12% out of the whole cohort was double positive for Sema4D and PD-L1 (Table 2) (Figure 4), and that PD-L1 correlated directly with Sema4D^+ve/high^ TAIs (p=0.007) (Table 3). The stratification of HNSCC based on combined PD-L1/Sema4D expression can open new avenues in addressing anti-PD-L1 resistant cases.

More importantly, in this study is the high levels of Sema4D detected in the plasma of HNSCC patients compared to healthy donors (Figure 5). Peripheral blood allowed for better healthy/normal control, as the tumor tissue controls were inflamed and positive for Sema4D (Supplementary Table 1) (Table 4). Future studies in which Sema4D levels in plasma can be paired with stromal phenotype and level of expression in tumor cells, can be used to validate the potential of Sema4D as a stromal biomarker in the peripheral blood [88]. Our data showed statistically insignificant higher levels of Sema4D in plasma of females compared to males. This might be due to the fact the estrogen was reported to increase shedding of Sema4D from platelets [10]. The finding that the autoimmune collagenous disease, Rheumatoid arthritis, and the Asthma patients with allergic inflammatory profile, showed comparable levels of Sema4D in plasma as HNSCC patients, suggests that Sema4D levels are associated with inflammation, rather than being specific to HNSCC. That Sema4D as an inflammatory mediator, can serve as a promising prognostic and monitoring biomarker for underlying inflammatory profile of HNSCC patients [89]. The association of Sema4D with malignancy is a matter of relative increase in the level of expression, where our cut off value for the examined tissue was diffuse strong expression (75%-100%), by the tumor cells or TAIs. The classical example in this context is the EGFR receptor, where the prognostic value is in the quantitative assessment, as it is present in normal epithelial tissue [90].

Intriguingly, allergy and autoimmunity are the main toxic tumor immunotherapy effects [91]. The fact that Sema4D is upregulated in tumor tissue as well as in autoimmune and allergic conditions, renders inhibition of Sema4D very promising, not only for immunotherapy resistant patients, but also for potential taming of the immune system, to decrease toxic immunotherapy effects.

The current work, presents a novel stratification model of HNSCC based on differential expression of Sema4D and PD-L1. We also present Sema4D as a novel biomarker for the peri-tumoral stromal phenotype. These findings open new avenues for targeted inhibition of Sema4D as a monotherapy or in combination regimen in HNSCC, that can achieve an inflammatory permissible stroma and set the stage for drug trafficking and several therapeutic modalities.

## MATERIALS AND METHODS

### HNC tissue microarray

Formalin-fixed, paraffin-embedded tissue microarray of head and neck cancer (HN802, HN803c and HN801b) was obtained from US Biomax (Rockville, MD). Demographic information, clinical stage, and pathological grade were provided. Normal to inflamed tissue sections from the tongue, and epiglottis and salivary glands were included as normal controls in the TMA. The hematoxylin and eosin sections were examined by an oral and maxillofacial pathologist to verify histopathological diagnosis prior to immunohistochemical (IHC) staining.

### Plasma samples

Plasma of 33 HNSCC patients and 5 rheumatoid arthritis (RA) patients, was collected under an approved protocol by the Institutional Review Board at the University of Maryland School of Medicine. Banked HNSCC and RA patient samples were obtained from patients with their written and informed consent. Plasma of 10 healthy donors, 5 of asthma patients and extra five of HNSCC patients were purchased from Innovative research (Novi, MI).

### Immunohistochemistry and immunofluorescence

For Sema4D staining, the avidin-biotin complex (ABC) technique was used following Vectastain elite ABC kit (PK-6102, mouse IgG) (Vector Laboratories, CA). Briefly, tissue sections were deparaffinized in xylene, rehydrated in graded ethanol, treated with Tris-EDTA buffer for antigen retrieval, and quenched in hydrogen peroxide to block endogenous peroxidase. Tissue sections were blocked with 2.5% normal plasma, incubated overnight at 4^°^C with anti- Sema4D antibody 1:200, (clone 30/CD100; Catalog no. 610670) (BD Transduction Laboratories),followed by biotinylated secondary antibody (catalog no. BA-9200), then the ABC reagent. Mouse IgG1, k isotype control antibody, was used (catalog No 400101)(Biolegend, San Diego, CA) Diaminobenzidine (SK-4105) was used as chromogen and counterstained with Mayer’s hematoxylin (Sigma-Aldrich Corp.). The Sema4D labeling index (LI) reflecting the intensity and extent of staining in the tumor cells was defined semiquantitatively as (0, 1, 2, and 3) and digitally using the Aperio software (Positive Pixel count v9) Blue, yellow, orange and red. Where (0) was negative (Blue digitally), (1) was diffuse weak staining (yellow digitally), (2) diffuse weak positivity with scattered strong positive <25% (yellow with scattered orange digitally), and (3) was for diffuse strong positivity or focal strong positive >25% (orange and red digitally). Since Sema4D showed negative to weak expression with focal strong positivity in the inflamed epithelium (Supplementary Figure 2), the cut off value semi quantitatively for Sema4D^+ve/high^ in tumor cells was the score (3), and digitally was total strong pixel intensity of ≥ 5x 10^5^ (diffuse orange to red), calculated as the median value between Sema4D^-ve/low^ and Sema4D ^+ve/high^ expression (Supplementary Figure 4). For PDL-1 staining, PD-L1 primary antibody clone SP142 (Roche/Ventana medical system, Inc) was used following the vendor recommended protocol. Since PD-L1 was negative in normal epithelium, the cut off value for PD-L1^+ve/high^ was strong focal expression ≥1% in tumor cells or in TAIs or both [84], and using the same digital cutoff value as with Sema4D. The strong pixel intensity for all the stains was calculated as the mean of three representative annotation layers of ∼0.2 mm^2^ area at 20x magnification, 200um scale.

Stromal analysis for fibrosis or TAIs infiltrate was carried out by OMP pathologist on the H&E and IHC stained sections under light microscopy. For analysis of fibrosis in the tumor stroma, delicate fibrous stroma (+), moderately fibrotic (++) and densely fibrotic (+++) parameters were used. The cut off value for dense fibrotic stroma was (+++). For the analysis of the tumor associated inflammatory cells (TAIs), (0, 1, 2, and 3) LI was used to reflect the extent of infiltrate of Sema4D^+ve/high^ TAIs in the peri-tumoral stroma. Where (0) was stroma completely negative, (1) was mild, (2) moderate and (3) for heavy infiltrate. The cut off value for the extent of infiltrate of Sema4D^+ve/high^ TAIs was the score (2). Photos were taken using the Aperio ScanScope and Image Scope software.

For immunofluorescence (IF) the TMA was processed through the same steps as for the IHC. Primary antibodies, anti- Sema4D antibody (clone 30/CD100) and CD163 (clone SP96, Catalog no. SAB5500042) (Sigma Aldrich,St. Louis, MO) were used and detected using the secondary antibodies anti-mouse IgG Alexa Fluor 488 (catalog no. A-11001) and anti-rabbit Alexa Fluor 546 (catalog no. A11035) (Thermo Fischer, Rockford, IL), respectively. The tissue was viewed using LSM DUO confocal microscope at the center for innovative biomedical resources core facility, University of Maryland Medical center.

### Picro Sirius tissue staining and *in vitro* fibrosis assay

Picro Sirius red stain (Catalog no. ab150681) (Abcam, Cambridge, MA) was used to stain HNSCC on TMA HN801b to demonstrate the collagenous peri-tumoral stroma, following the manufacturer procedures. The tissue was scanned using the Leica biosystem Aperio Scanscope and Photos were taken using the Image Scope software. For viewing type I and type III collagen, polarizing lenses were used on Alpha and Omega bright light microscope and photos were taken using attached infinity capture software, 20MPX camera. For the *in vitro* fibrosis assay, fibroblasts were grown in 96 well plate for 72hrs in CM obtained from WSU-WSU-HN6 repleted with 4%FBS and 1% PSA. Then cells were fixed in methanol overnight in -20°C, then washed once with PBS, followed by incubation in the Picro Sirius red stain for 1 hour in room temperature. The stain was eluted in 0.1 N sodium hydroxide 100ul/well on a rocking platform at room temperature for 1 hour, and then read at 540nm using BioTek Epoch microplate spectrophotometer.

### Sema4D and TGF-β1 ELISA

Sema4D concentration in the plasma was determined using direct ELISA. Immulon 4 HBX microtiter plates (Thermo Scientific, Waltham, MA) were coated with 50 microliters of undiluted plasma, washed, then incubated with anti-human CD100 antibody (clone: 133-1C6; Novus Biologicals). Goat anti-mouse IgM-HRP (catalog no. M31507; Life Technologies) was added followed by detection with TMB (Pierce). The plasma concentrations of Sema4D were calculated using the standard curve established for recombinant Sema4D (catalog no. 310-29) (Peprotech, RockyHill, NJ). The detection limit was 3.1 ng/mL. Mouse IgM isotype control was used for the direct ELISA assay (Catalog no. ab91546) (Abcam). MYBioSourceHuman Semaphorin sandwitch ELISA kit (Catalog no. MBS763518) was used to confirm the direct ELISA results. For detection of TGF-β1 by WSU-WSU-HN6 tumor cells, Human ELISA TGF-β1 total kit was used following manufacturer recommendations (catalog no.436707) (BioLegend, San Diego, CA). Conditioned medium was run in triplicates and plates were read using BioTek Epoch microplate spectrophotometer at 450nm wavelength.

### Tissue culture and cell lines

The oral squamous cell carcinoma cell lines WSU-HN6 (T3N2bM0), and WSU-HN13 (T2N2M0), and the normal oral keratinocytes (NOK) normal control cell line, were DNA authenticated at Johns Hopkins Genetic Resources Core Facility, Baltimore, MD, to ensure consistency in cell identity in comparison with their source [54]. The WSU-HN4 a stage IV (T4N1M0) malignancy of the base of the tongue was a gift from Dr. Silvio Gutkind (The National Institute of Dental and Craniofacial research, Bethesda, MD). SCC-9 (ATCC® CRL-1629) and the human dermal fibroblasts (ATCC® PCS-201-012TM) are from American Type Culture Collection (Manassas, VA). All HNSCC cell lines used were derived from primary carcinomas of the tongue. All cell lines were grown and maintained in DMEM supplemented with 10% FBS (20% FBS for fibroblasts), 100 U/ml penicillin, 100 mg/ml streptomycin, and 250 ng/ml amphotericin B (PSA) (Sigma-Aldrich, St. Louis, MO) at 37°C in humidified air with 5% CO2. For treatment with conditioned medium, culture medium was collected from confluent HNSCC cells grown in 5 ml DMEM for 24 h.

### siRNA, shRNA and lentivirus infections

For Plexin-B1 and Sema4D gene silencing, the siRNA system Hs_PLXNB1_6 (Catalog no. SI02757566), Hs_SEMA4D_6 (Catalog no. SI03053701) were used respectively, with the HiPerFect transfection reagent (Catalog no.301704) (Qiagen Inc, Germantown, MD). Sema4D knockdown using shRNA lentivirus system was also used. The lentivirus and shRNA system were a gift of Dr. John R. Basile (University of Maryland, Baltimore) [24]. In brief, the shRNA sequences for human Sema4D were obtained from Cold Spring Harbor Laboratory’s RNAi library (RNAi Codex; http:katahdin.cshl.org:9331_homepage_portal_scripts_main2.pl). The oligonucleotides (Invitrogen, Grand Island, NY) used to knockdown Sema4D protein levels were 5’-GGCCTGAGGACCTTGCAGAAGA-3’. The Sema4D shRNA oligonucleotides were cloned into lentiviral expression vector pWPI GW as previously described (32). pWPI (empty) vector was used as negative control. Transductions were performed using FuGENE HD transfection reagent (catalog no. E2311; Promega). Cells were selected using 750ug/ml G418 and Knock down was verified using western blot.

### Immunoblots and antibodies

Cells were harvested in SDS cell lysis buffer with the addition of a protease inhibitor tablet (catalog no. 11836170001) (Roche Diagnostics, Indianapolis, IN). Whole-cell lysate was separated using SDS-PAGE. The primary Abs Sema4D (30/CD100, catalog no. 610670) (BD Biosciences/Pharmingen,San Diego, CA), Plexin-B1 ((A-8): sc-28372), GAPDH (8C2; sc-81545), (Santa Cruz Biotechnology, Dallas, TX) were used. Phospho ERK (Cat # 4370) and total ERK (Cat #4695) (Cell Signaling Technology, Danvers, MA) (The secondary Abs used were anti-rabbit IgG (catalog no. sc-2301) and anti-mouse IgG (catalog no. sc-2302) (Santa Cruz Biotechnology).

### Statistical analysis

Frequency distributions were estimated and compared using the Fisher’s Exact test. Due to rather small sample sizes the non-parametric Exact Wilcoxon test and Kruskal-Wallis test were also utilized to compare Sema4D level across patients’ categories (HNSCC, HD, RA), and to assess plausible effect of location, gender, race, clinical stage, and lymph node metastases on Sema4D levels in plasma of HNSCC patients. General linear models approach was used to compare means for continuous variables. All reported p-values are nominal, 2-sided, and exact. Testing was done at 0.05 level of significance. Statistical analysis was conducted using SAS (v.9.4, SAS Inc. Cary, NC)

For all *in vitro* studies, student paired t tests were performed as appropriate. All data analysis was presented with custom SD. The p values are ^*^p ≤ 0.05; ^**^p ≤0.01; ^***^p ≤0.001.

## SUPPLEMENTARY MATERIALS FIGURES AND TABLES


