# Supplementary material for: Semaphorin 4D in human head and neck cancer tissue and peripheral blood: A dense fibrotic peri-tumoral stromal phenotype
Source: Oncotarget. 2018 Jan 19;9(13):11126–44. doi: 10.18632/oncotarget.24277 (PMC5834246; doi:10.18632/oncotarget.24277)
Supplement: Supplementary file 1 [file oncotarget-09-11126-s001.pdf]

Semaphorin 4D in human head and neck cancer tissue and peripheral blood: A dense fibrotic peri-tumoral stromal phenotype

SUPPLEMENTARY MATERIALS

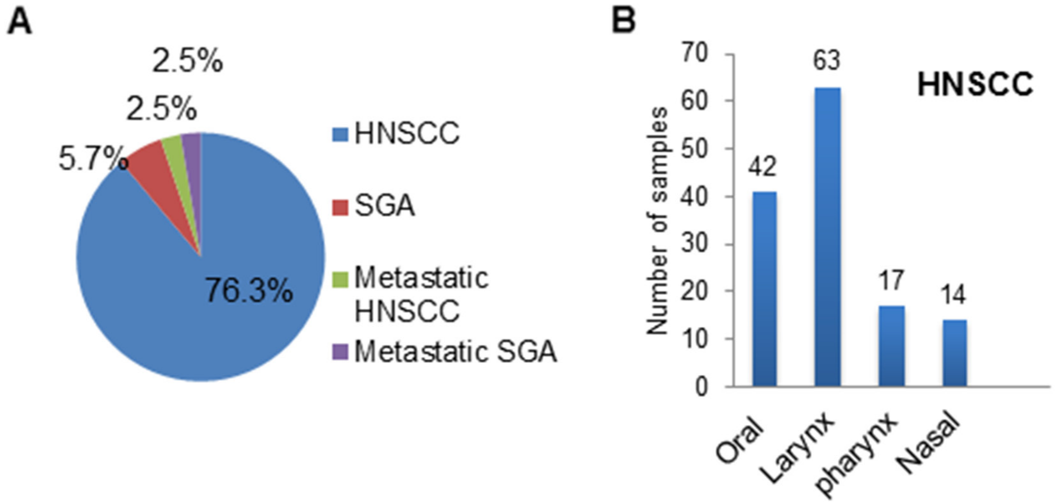

Supplementary Figure 1: Graphical presentation of the human HNC tumor samples. **(A)** Pie chart representative of the HNC tumors included in the study. **(B)** Graphical presentation of the HNSCC cases included in the study according to tissue of origin. HNC; Head and Neck Cancer, HNSCC; Head and Neck Squamous cell carcinoma, SGA; Salivary gland adenocarcinoma.

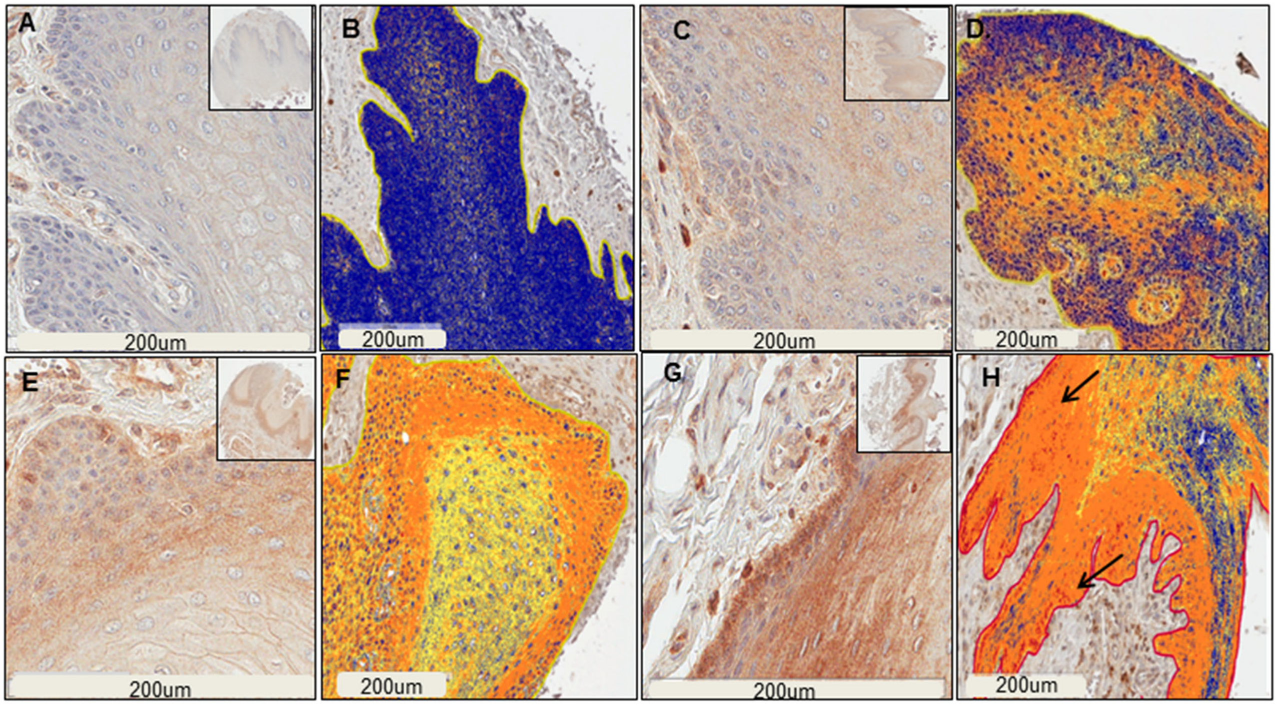

Supplementary Figure 2: Sema4D expression in the normal epithelium and epithelial tissue adjacent to tumor margin. **(A)** Normal parakeratinized stratified squamous epithelium of the tongue is negative for Sema4D expression. **(B)** Digital analysis of tissue in (A) showing negative Sema4D expression (blue) through the entire thickness with only $6x10^3$ strong positive pixel intensity. **(C)** Parakeratinized stratified squamous epithelium showing Sema4D$^{-ve/low}$ expression with focal positivity in the prickle cells. The inflammatory cells in the stroma are positive for Sema4D. **(D)** Digital analysis of tissue in (C) shows negative (blue) to focal positive (orange) expression of the prickle cell layer with $3.5\ x10^4$ of strong positive pixel intensity. **(E)** hyperkeratotic Tongue epithelium showing focal Sema4D$^{+ve/high}$ expression in the parabasal cell layer. **(F)** Digital analysis of (E) shows diffuse weak expression (yellow) in the prickle cells, to focal positive (orange) expression in the parabasal layer with $2x10^5$ mean of strong positive pixel intensity. **(G)** Sema4D$^{+ve/high}$ in the basal and prickle cell layer of the surface epithelium (arrows). **(H)** Digital analysis of Sema4D staining in (G) shows high (red) expression in the basal cell layer and diffuse positive expression in the prickle cell layer with $1.3x10^6$ mean of strong positive pixel intensity.The strong positive pixel intensity presented is the mean of three annotations of each tissue section. (20x; 200um scale, in set: 40x). Cut off for positive expression is ($5x10^5$)

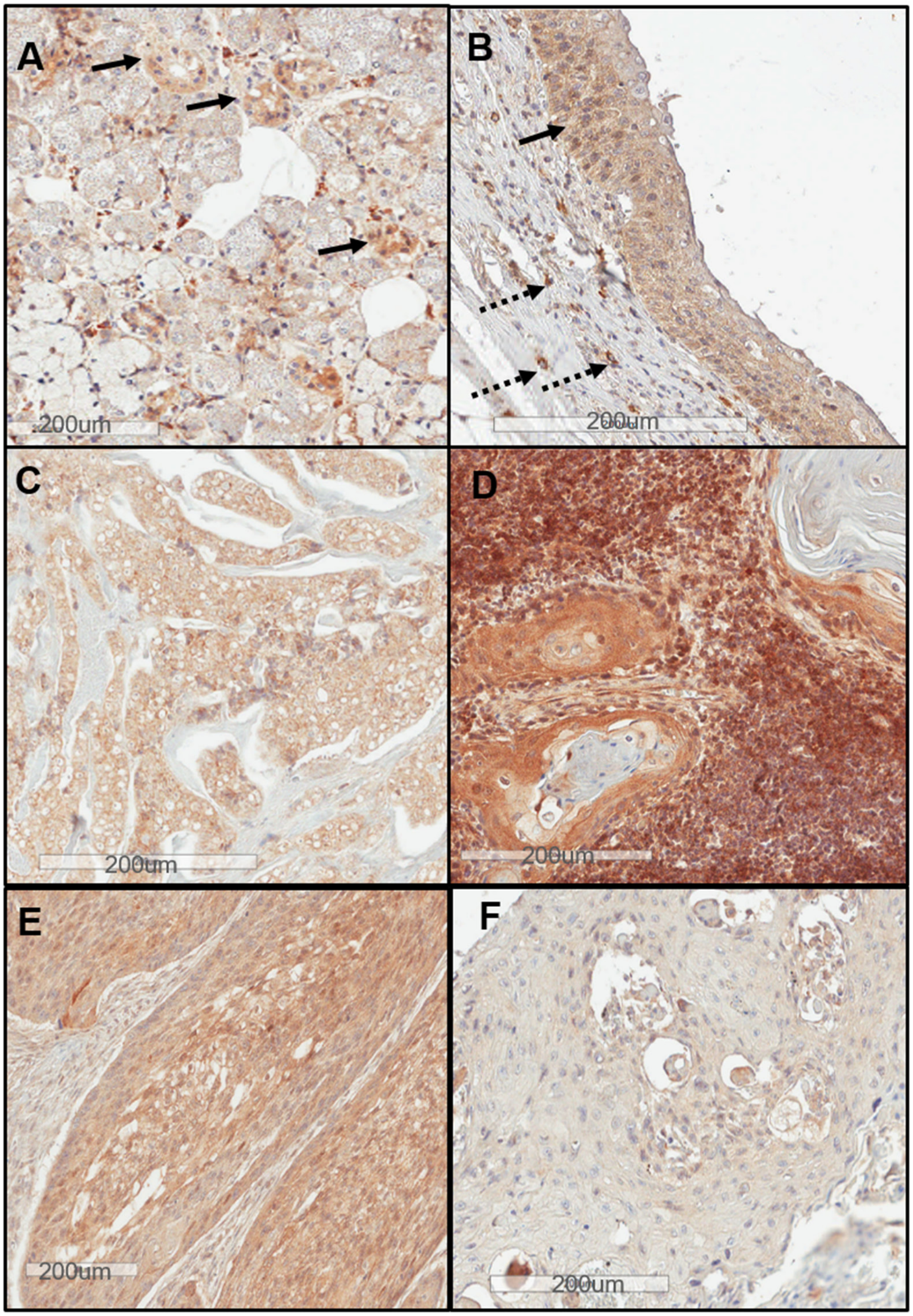

Supplementary Figure 3: Sema4D expression in normal salivary gland tissue and epiglottis. **(A)** Normal salivary gland acini and ducts. Positive expression in the ductal cells (arrows). Mucous and serous acini are negative. **(B)** Inflamed epiglottis shows weak positivity to Sema4D expression in the lining epithelium (arrow) and focal expression in inflammatory cells in the stroma (dotted arrow) (20x; 200um scale). Sema4D expression in salivary adenocarcinoma and metastatic tumors. **(C)** Adenoid cystic carcinoma of the parotid gland shows diffuse strong Sema4D expression in tumor cells and hyalinized stroma. **(D)** Metastatic SCC to the lymph nodes. Tumor cells show strong Sema4D$^{+ve/high}$ expression as well as the inflammatory cells of the lymph node. **(E)** Primary laryngeal SCC stage T3N1M0, Tumor cells are Sema4D $^{+ve/high}$. **(F)** Primary SCC of the tongue stage T1N0M0, tumor cells shows Sema4D$^{-ve/low}$. (20x; 200um scale)

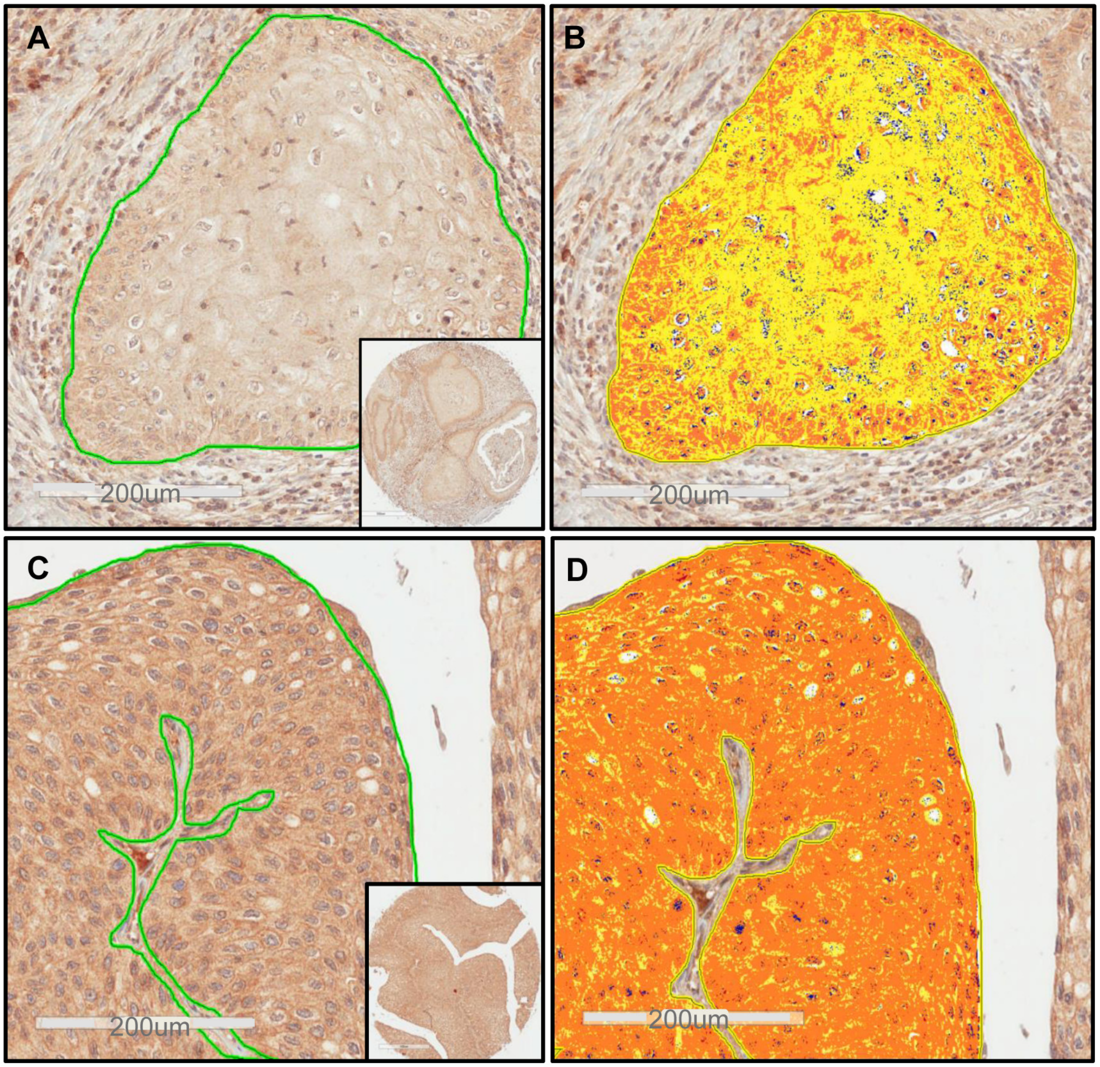

Supplementary Figure 4: Standardization of Sema4D expression in tumor islands. **(A)** Stage I SCC of the lip showing$^{-ve/low}$ expression of Sema4D in the annotated tumor island. **(B)** Digital analysis of Sema4D staining in (A) showing low expression (yellow), in the tumor cells with low level of strong positive pixel intensity of $1.5x10^5$. **(C)** Stage III SCC of the left upper jaw, showing diffuse Sema4D$^{+ve/high}$ expression, with annotation set for digital analysis. **(D)** Digital analysis of Sema4D expression in (C) showed high level of strong positive pixel intensity of $1.1x10^6$. The total intensity of strong positive pixels was calculated as the mean of three different annotations representative of the whole tumor, each annotation was analyzed under 200x magnification in an area of ~ 0. 2 mm^2 (20x; 200um scale, inset: 40x). Cut off for positive expression is ($5x10^5$)

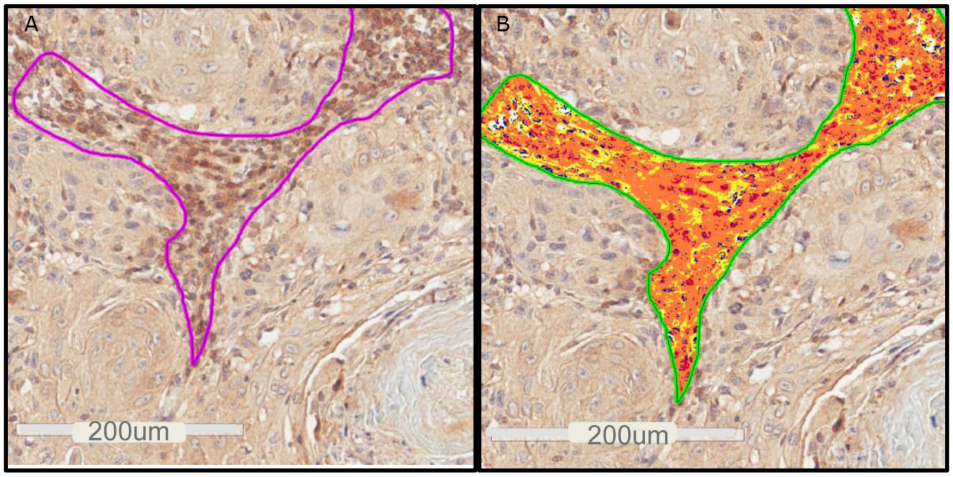

Supplementary Figure 5: Sema4D$^{+ve/high}$ TAIs. **(A)** TAIs infiltrating the stroma of stage II SCC of the right palate were Sema4D$^{+ve/high}$. **(B)** Digital analysis shows high Sema4D expression (red) in the TAIs with 1.3x10^{6} strong positive pixel intensity (20x; 200um scale). Cut off for positive expression is (5x10^{5}).

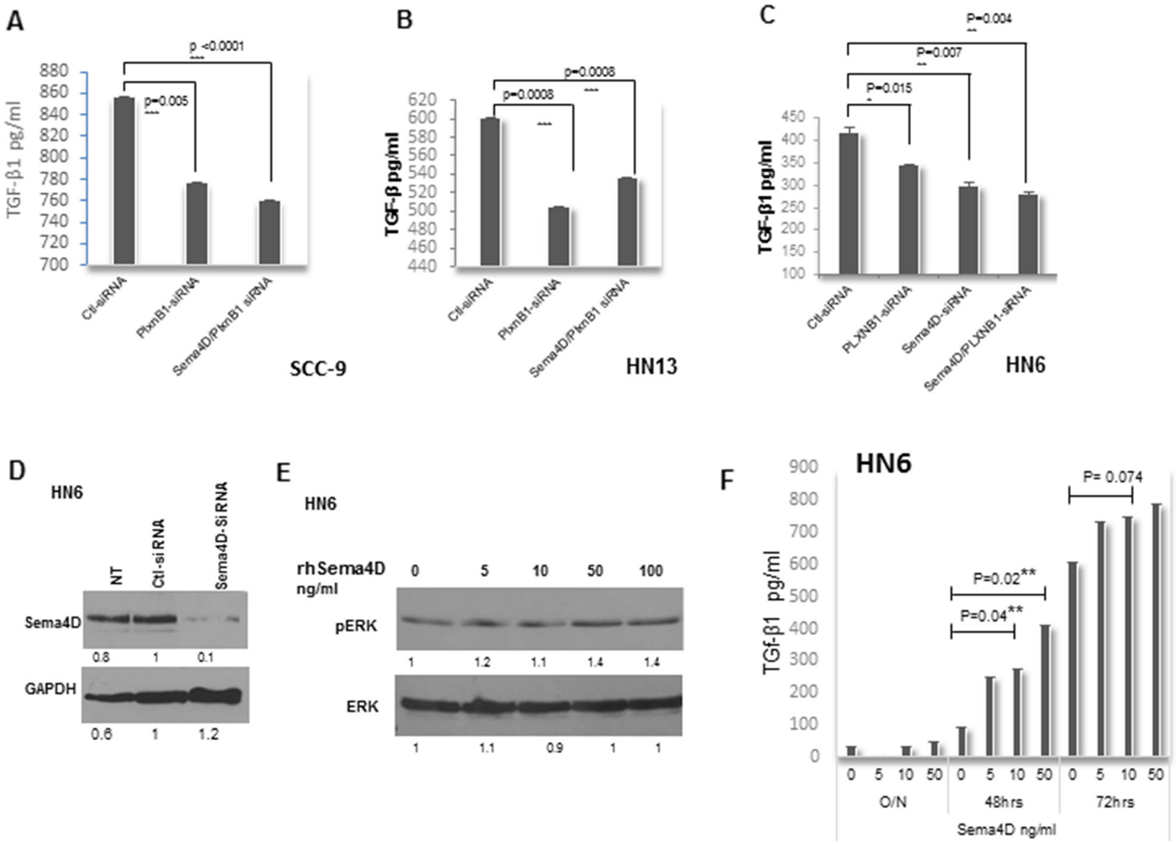

Supplementary Figure 6: Levels of activated TGF-β1 in CM. Decreased level of activated TGF-β1 in CM, **(A)** upon siRNA silencing of Plexin-B1, or combined Plexin-B1/Sema4D siRNA in SCC-9. **(B)** HN13 at 48hrs. **(C)** and HN6 at 72hrs. **(D)** Immunoblot of Sema4D siRNA in HN6. **(E)** Treatment ofHN6 with rhSema4D for 20 minutes shows evidence of increased ERK phosphorylation. **(F)** Levels of activated TGF-β1 in CM of HN6 upon treatment with rhSema4D. TGF-β1 levels in CM starting at 48hrs. PLXNB1:Plexin-B1, rhSema4D: recombinant human Sema4D, O/N: overnight, CM: culture media. Gel band quantified using *image J*.

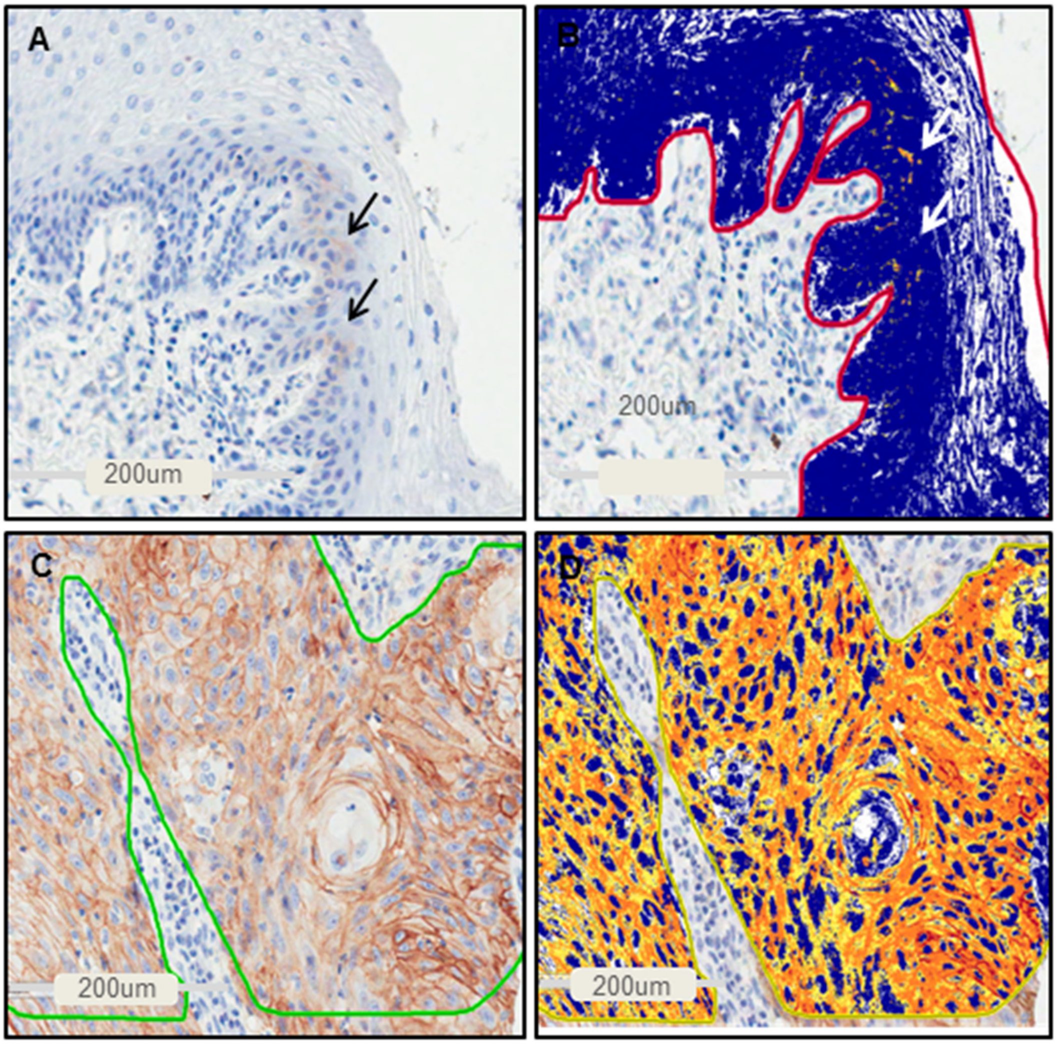

Supplementary Figure 7: PD-L1 expression in epithelial tissue adjacent to tumor margin and in tumor cells. (A) PD-L1 is negative in tissue adjacent to tumor margin with a focal positive area (arrows). **(B)** Digital analysis shows negative expression (blue) with focal positivity (arrows) (20x; 200um scale). **(C)** Tumor cells of stage II SCC of the right palate shows PD-L1 $^{+ve/high}$ expression in tumor cells, TAIs are PD-L1 negative. **(D)** PD-L1 staining in (C) showed high level of strong positive pixel intensity of 5.5 x10^6 (20x; 200um scale).

Supplementary Table 1: Descriptive analysis of Sema4D staining according to tissue Type

Tissue Type and distribution	Sema4D expression		Total	P value Fisher's Exact Test, 2-sided
	-ve/low (%)	+ve/high (%)		
HNSCC tumor cells	90 (66)	46 (34)	136 (100)	0.80
Control inflamed epithelium	11 (65)	6 (35)	17 (100)	
**Total**	101 (66)	52 (34)	153 (100)	
TAIs in HNSCC	49 (37)	82 (63)	131 (100)	0.58
Inflammatory Cells in control inflamed epithelium	7 (47)	8 (53)	15 (100)	
**Total**	56 (38)	90 (62)	146 (100)	

Supplementary Table 2: Sema4D expression in tumor cells in correlation to Patient's demographics and histological grade

	Sema4D expression in tumor cells		Total	P value
	-ve/low No (%)	+ve/high No (%)		
**Age**	90 (66)	46 (34)	136 (100)	
Median (Range)	57 (12-90)	59 (42-83)		0.31
**Gender**				
F	14 (64)	8 (36)	22 (16)	0.78
M	76 (67)	38 (33)	114 (34)	
Total	90 (66)	46 (34)	136 (100)	
**Location**				
Oral cavity	34 (83)	7 (17)	41 (30)	0.03
Pharynx	8 (47)	9 (53)	17 (13)	
Larynx	40 (63)	23 (37)	63 (47)	
Nasal cavity	8 (57)	6 (43)	14 (10)	
Total	90 (67)	45 (33)	135 (100)	
**SCC Hist grade**				
1	34 (72)	13 (28)	47 (35)	0.15
2	33 (57)	25 (43)	58 (44)	
3	21 (75)	7 (25)	28 (21)	
Total	88 (66)	45 (34)	133 (100)	

Supplementary Table 3: Sema4D$^{+ve/high}$ TAIs in correlation toPatients' demographics and clinical staging

	TAIs expressing Sema4D		Total	P value
	-ve/low N (%)	+ve/high N (%)		
**Age**	49 (37)	83 (63)	132 (100)	
Median (range)	56 (41-90)	57 (12-81)		0.57
**Gender**				
M	43 (38)	69 (62)	112 (85)	0.47
F	6 (30)	14 (70)	20 (15)	
Total	49 (37)	83 (62)	132 (100)	
**Location**				
Oral Cavity	11 (29)	27 (71)	38 (29)	0.58
Larynx	26 (41)	37 (59)	63 (48)	
Pharynx	5 (31)	11 (69)	16 (12)	
Nasal cavity	6 (43)	8 (57)	14 (11)	
Total	48 (37)	83 (63)	131 (100)	
**Grade**				
1	14 (31)	31 (69)	45 (35)	0.35
2	21 (37)	36 (63)	57 (44)	
3	13 (48)	14 (52)	27 (21)	
Total	48 (37)	81 (63)	129 (100)	
**Tumor stage**				
I+II	19 (34)	37 (66)	56 (43)	0.68
III	14 (37)	24 (63)	38 (30)	
IV	15 (43)	20 (57)	35 (27)	
Total	48 (37)	81 (63)	129 (100)	
**Nodal metastasis**				
Yes	15 (42)	21 (58)	36 (28)	0.51
No	33 (35)	60 (65)	93 (72)	
Total	48 (37)	81 (63)	129 (100)	

Supplementary Table 4: Descriptive analysis of PD-L1 staining according to tissue type

Type	PD-L1 expression		Total	P value Fisher's Exact Test, 2-sided
	-ve/low No (%)	+ve/high No (%)		
HNSCC tumor cells	99 (73)	36 (27)	135 (89)	0.07
Normal/Inflamed epithelium	16 (94)	1 (6)	17 (11)	
Total	115 (76)	37 (24)	152 (100)	
**HNSCC tumor cells and/or TAIs**	86 (64)	49 (36)	135 (89)	0.01
Normal/Inflamed epithelium	16 (94)	1 (6)	17 (11)	
Total	102 (67)	50 (33)	152 (100)	

Supplementary Table 5: TotalPD-L1 expression in correlation to patients' demographics

	PD-L1 expression in tumor tissue		Total	P-value
	-ve/low N (%)	+ve/high N (%)		
**Age**	86 (63)	49 (37)	135 (100)	0.15
Median (range)	57 (12-83)	60 (38-90)		
**Gender**				
Males	70 (63)	42 (37)	112 (84)	0.80
Females	15 (68)	7 (32)	22 (16)	
Total	85 (63)	49 (37)	134 (100)	
**Location**				
Oral cavity	23 (56)	18 (44)	41 (31)	0.25
Larynx	40 (65)	22 (35)	62 (46)	
Pharynx	10 (59)	7 (41)	17 (13)	
Nasal cavity	12 (86)	2 (14)	14 (10)	
Total	85 (63)	49 (37)	134 (100)	
**Pathological grade**				
1	31 (67)	15 (33)	46 (35)	0.46
2	37 (65)	20 (35)	57 (44)	
3	15 (54)	13 (46)	28 (21)	
Total	83 (63)	48 (37)	131 (100)	
